# IFNAR signaling of neuroectodermal cells is essential for the survival of C57BL/6 mice infected with Theiler’s murine encephalomyelitis virus

**DOI:** 10.1186/s12974-023-02737-6

**Published:** 2023-03-05

**Authors:** Melanie Bühler, Dandan Li, Lin Li, Sandra Runft, Inken Waltl, Andreas Pavlou, Ulrich Kalinke, Malgorzata Ciurkiewicz, Jochen Huehn, Stefan Floess, Andreas Beineke, Wolfgang Baumgärtner, Ingo Gerhauser

**Affiliations:** 1 Department of Pathology, University of Veterinary Medicine Hannover, Foundation, Bünteweg 17, 30559 Hannover, Germany; 2 Centre for Systems Neuroscience (ZSN), Hannover, Germany; 3 c/o School of Basic Medical Sciences, Shanxi Medical University, Shanxi, China; 4 Institute for Experimental Infection Research, TWINCORE, Centre for Experimental and Clinical Infection Research, a joint venture between the Helmholtz Centre for Infection Research and the Hannover Medical School, Hannover, Germany; 5 Experimental Immunology, Helmholtz Centre for Infection Research, Inhoffenstraße 7, 38124 Brunswick, Germany

**Keywords:** Bead-based immunoassay, Cell-type specific knockout mice, Cytokine, Immunohistochemistry, RNA-seq-analysis, RT-qPCR, Theiler’s murine encephalomyelitis virus, Type I interferon receptor, Viral encephalitis

## Abstract

**Background:**

Theiler’s murine encephalomyelitis virus (TMEV) is a single-stranded RNA virus that causes encephalitis followed by chronic demyelination in SJL mice and spontaneous seizures in C57BL/6 mice. Since earlier studies indicated a critical role of type I interferon (IFN-I) signaling in the control of viral replication in the central nervous system (CNS), mouse strain-specific differences in pathways induced by the IFN-I receptor (IFNAR) might determine the outcome of TMEV infection.

**Methods:**

Data of RNA-seq analysis and immunohistochemistry were used to compare the gene and protein expression of IFN-I signaling pathway members between mock- and TMEV-infected SJL and C57BL/6 mice at 4, 7 and 14 days post-infection (dpi). To address the impact of IFNAR signaling in selected brain-resident cell types, conditional knockout mice with an IFNAR deficiency in cells of the neuroectodermal lineage (NesCre^±^IFNAR^fl/fl^), neurons (Syn1Cre^±^IFNAR^fl/fl^), astrocytes (GFAPCre^±^IFNAR^fl/fl^), and microglia (Sall1Cre^ER±^IFNAR^fl/fl^) on a C57BL/6 background were tested. PCR and an immunoassay were used to quantify TMEV RNA and cytokine and chemokine expression in their brain at 4 dpi.

**Results:**

RNA-seq analysis revealed upregulation of most ISGs in SJL and C57BL/6 mice, but *Ifi202b* mRNA transcripts were only increased in SJL and *Trim12a* only in C57BL/6 mice. Immunohistochemistry showed minor differences in ISG expression (ISG15, OAS, PKR) between both mouse strains. While all immunocompetent Cre-negative control mice and the majority of mice with IFNAR deficiency in neurons or microglia survived until 14 dpi, lack of IFNAR expression in all cells (IFNAR^−/−^), neuroectodermal cells, or astrocytes induced lethal disease in most of the analyzed mice, which was associated with unrestricted viral replication. NesCre^±^IFNAR^fl/fl^ mice showed more *Ifnb1*, *Tnfa*, *Il6*, *Il10*, *Il12b* and *Ifng* mRNA transcripts than Cre^−/−^IFNAR^fl/fl^ mice. IFNAR^−/−^ mice also demonstrated increased IFN-α, IFN-β, IL1-β, IL-6, and CXCL-1 protein levels, which highly correlated with viral load.

**Conclusions:**

*Ifi202b* and *Trim12a* expression levels likely contribute to mouse strain-specific susceptibility to TMEV-induced CNS lesions. Restriction of viral replication is strongly dependent on IFNAR signaling of neuroectodermal cells, which also controls the expression of key pro- and anti-inflammatory cytokines during viral brain infection.

**Supplementary Information:**

The online version contains supplementary material available at 10.1186/s12974-023-02737-6.

## Background

Theiler’s murine encephalomyelitis virus (TMEV) belongs to the genus Cardiovirus of the *Picornaviridae* family. Members of the Theiler's original (TO) group of TMEV (BeAn and DA) cause a biphasic demyelinating disease after intracranial injection into the central nervous system (CNS) of susceptible mouse strains such as SJL [1]. In contrast, resistant mouse strains including C57BL/6 mice only exhibit acute encephalitis but clear the virus from the CNS [1–3]. The susceptibility to TMEV-induced demyelinating disease (TMEV-IDD) is partly controlled by the major histocompatibility I complex (MHC-I) H-2D region and several non-H2 loci such as Tmevp3, which contains the interferon (IFN)-γ gene [4, 5]. Furthermore, a recent study using RNA-based next generation sequencing (RNA-seq) revealed more than 700 differentially expressed genes between C57BL/6 and SJL mice after TMEV infection [6]. Functional categories of these genes included the terms antigen processing and presentation, response to cytokine, and response to interferon (IFN)-β. In accordance with this, TMEV-infected IFN-β^−/−^ C57BL/6 mice show an impaired virus elimination capacity and 70% of these mice develop mild demyelination [7]. IFNs are cytokines that belong to the first line of antiviral response of the innate immune system [8, 9]. Type I IFNs (IFN-I) including IFN-α and -β interfere with viral replication, whereas the type II IFN IFN-γ represents a strong activator of macrophages and stimulator of type 1 T helper (T_H_1) cells-mediated immune reactions [10]. Most importantly, IFN-I induces a wide range of IFN-stimulated genes (ISGs) via the JAK–STAT pathway mediating pleiotropic effects [11–13].

A higher constitutive expression of IFN-stimulated protein of 15 kDa (ISG15), protein kinase RNA-activated (PKR) and 2′5′-oligoadenylate synthetase (OAS) was described in the spinal cord of C57BL/6 compared to SJL mice [14]. Moreover, C57BL/6 mice show a higher number of *Rnasel* mRNA transcripts in the brain than SJL mice [6]. ISG15 is a ubiquitin-like protein that can inhibit virus production and modify the function of cellular proteins by a process called ISGylation [15]. PKR is activated by double-stranded (ds) RNA and inhibits viral replication through the phosphorylation of eukaryotic translation initiation factor 2A (eIF2) [16, 17]. The OAS/RNase L pathway is also activated by dsRNA and blocks viral infection through cleavage of viral and cellular RNA [18]. Nevertheless, the leader (L) protein of TMEV prevents the activation of PKR via an indirect mechanism and directly blocks the OAS/RNase L pathway [19–21]. In addition, the L protein inhibits the cellular mRNA export from the nucleus and blocks IFN-I transcription by inhibition of IFN-regulated factor (IRF)-3 dimerization [22, 23]. Consequently, TMEV impedes the expression of IFN-I and the function of several ISGs including OAS and PKR, but might not prevent direct antiviral effects of other ISGs such as ISG15, which is strongly expressed by astrocytes and endothelial cells [14]. However, the knowledge about the specific role of CNS-resident cells in IFN-I signaling pathways upon TMEV infection remains minimal.

During acute encephalitis, TMEV can be detected predominantly in astrocytes and neurons, but microglia/macrophages, oligodendrocytes and ependymal cells can be infected as well [24, 25]. The protection against TMEV-IDD depends on the induction of TLR-3- and MDA-5-mediated signaling pathways, which induce the production of IFN-I and proinflammatory cytokines [26, 27]. Several in vitro and in vivo studies investigated the contribution of CNS-resident cells to IFN-I production in infectious and autoimmune diseases [28–30]. IFN-I is expressed at low basal levels in the normal CNS and is crucial for synaptic plasticity, cognitive function and neurodegeneration [31–34]. Productively infected neurons contribute to IFN-I production while abortively infected astrocytes are the main source of IFN-I after infection of the brain with rabies virus, vesicular stomatitis virus, and TMEV. Interestingly, myeloid cells such as microglia seem to have minor contribution to IFN-I production during neuroinfection [29, 30]. Nevertheless, IFN-β produced by other cells than astrocytes seems to be sufficient to maintain the resistance of C57BL/6 mice against TMEV-IDD [7].

All CNS cell types including neurons can respond to IFN-I stimulation in vivo [34]. The use of IFN-I receptor knockout (IFNAR^−/−^) mice of the 129 Sv strain confirmed the prominent role of the IFN-I system in the innate immune response against TMEV [35, 36]. This inbred mouse strain is normally resistant to TMEV infection and clears the virus within days, but complete deficiency of IFNAR signaling results in rapid fatal encephalitis due to increased viral load and CNS inflammation [35, 36]. TMEV-infected IFNAR^−/−^ mice showed a less efficient stimulation of virus-specific CD4^+^ and CD8^+^ T cell responses, which is partially caused by a reduced expression of activation markers on antigen-presenting dendritic cells [36]. IFN-I also promotes the differentiation of CD4^+^ T cells into T_H_1 cells by increasing their IL-2 responsiveness and acts directly on CD8^+^ T cells to allow clonal expansion and memory formation in response to viral infection [37, 38]. Additionally, IFNAR signaling in astrocytes regulates the permeability of the blood–brain barrier during virus infections [39, 40].

The authors hypothesized that intrinsic differences in the innate immune system between SJL and C57BL/6 mice contribute to the differences in their susceptibility to TMEV-IDD. The aim of this study was (i) to compare the mRNA and protein expression of selected IFN-I pathway members (ISG15, OAS, PKR) between these two mouse strains after TMEV infection and (ii) to determine the relevance of IFN-I stimulation of astrocytes, neurons and microglia for the control of virus replication in vivo using cell-type specific IFNAR-deficient mice. The presented data demonstrate the prominent role of astrocytes and neurons in the coordination of the IFN-dependent innate immune response, which is necessary for the control of viral replication and cytokine expression.

## Methods

### Animal experiments

Initially, female SJL/JHanHsd and C57BL/6JOlaHsd mice were obtained from Harlan Winkelmann (Borchen, Germany) and intracerebrally infected with 1.63 × 10^6^ plaque-forming units (PFU) of TMEV-BeAn under deep anesthesia at the age of 5 weeks [6]. Mock-infected animals served as controls. Groups of six animals were euthanized with an overdose of medetomidine (Domitor®) and ketamine (Ketamin Gräub®) at 4, 7 and 14 days post-infection (dpi). Brain and spinal cord samples were either fixed in 10% formalin for histology and immunohistochemistry or embedded in OCT® embedding compound (Sakura Finetek Germany GmbH, Umkirch, Germany) for storage at -80 °C and RNA extraction [6].

Moreover, IFNAR-deficient (IFNAR^−/−^) mice were generated on a 129/Sv background [41] and 20-fold backcrossed to the C57BL/6 background [42]. Cell type-specific IFNAR^−/−^ C57BL/6 mice were obtained by intercrossing IFNAR^fl/fl^ mice [43] and transgenic mice that express Cre specifically in neuroectodermal cells (NesCre^±^) [44], neurons (Syn1Cre^±^) [45], astrocytes (GFAPCre^±^) [46] and microglia (Sall1Cre^ER±^) [47]. These mouse strains were tenfold backcrossed to the C57BL/6 background before intercrossing. Negative littermates (Cre^−/−^) were used as control animals. In order to induce Cre^ER^ activity in Sall1Cre^ER±^ IFNAR^fl/fl^ mice, 3- to 4-week-old animals were treated subcutaneously with 4 mg of tamoxifen (Tam; Sigma-Aldrich Chemie GmbH, Taufkirchen, Germany) 5 times with 48-h interval. Sall1Cre^ER−/−^ IFNAR^fl/fl^ mice also treated with Tam served as controls. At the age of 5–6 weeks, female and male complete and cell-type specific IFNAR-deficient C57BL/6 mice were intracerebrally infected with 1 × 10^5^ PFU of TMEV-BeAn strain under deep anesthesia [48]. Behavior and activity (0–3 points), outer appearance and posture (0–3 points) and gait (0–4 points) of these mice were evaluated semiquantitatively. Weight was measured weekly or daily when clinical signs appeared [48]. The points in each category were added up to the final clinical score. Mice were either euthanized when reaching the humane endpoint (maximal score in one category or final clinical score ≥ 8 points) or at 4 dpi or at 14 dpi. Brain samples were either formalin-fixed or snap-frozen for further analysis (see above).

All mice were kept in a microisolator cage system (Tecniplast, Hohenpeißenberg, Germany) with ad libitum access to food and water. Only female mice were used in the initial experiments to facilitate group housing of mice (3 mice per cage), which allows social interaction and improves animal welfare. In order to minimize the overall number of complete and cell-type specific IFNAR-deficient mice generated by the authors, female and male mice were included in the experiments based on the principles of the 3Rs (Replacement, Reduction and Refinement). All animal experiments were performed according to the German law of animal protection and authorized by local authorities (Niedersächsisches Landesamt für Verbraucherschutz und Lebensmittelsicherheit, Oldenburg, Germany, permission numbers: 509c-42502-02/589, 33-42502-05/963, 33.12-42502-04–14/1656).

### Histology and immunohistochemistry

2–4 µm paraffin coronal sections of the cerebrum at the level of the hippocampus, the cerebellum at the level of the cerebellar nuclei, and the cervical, thoracic and lumbar spinal cord were routinely stained with hematoxylin and eosin (HE). The number of perivascular mononuclear cells was quantified in the cerebrum of complete and cell-type specific IFNAR-deficient mice using a semiquantitative scoring system (0: no infiltrates; 1: one layer of infiltrates; 2: 2–3 layers of infiltrates; 3: > 3 layers of infiltrates). Eight areas (meninges, cortex cerebri, subcortical white matter, hippocampus, thalamus/hypothalamus, third ventricle, lateral ventricles and basal ganglia) were investigated separately in both hemispheres (16 values per animal) and the mean calculated for each mouse [7]. Moreover, hippocampal cell loss was semiquantitatively quantified (0: no neuronal loss; 1: loss of < 25% of neurons; 2: loss of 25–50% of neurons; 3: loss of > 50% of neurons).

Immunohistochemistry was performed as described [14, 49–51]. Briefly, after blocking of the endogenous peroxidase non-specific bindings sections were blocked with 20% goat or rabbit serum diluted in phosphate buffered saline. Then slides were either incubated with polyclonal rabbit antibodies directed against TMEV (capsid protein VP1, 1:2000) [49], CD3 (Agilent Dako, Santa Clara CA, USA; 1:1000), Iba-1 (019-19741, Wako Chemicals GmbH, Neuss, Germany; 1:200) GFAP (Z0334, Agilent Dako; 1:2000), ISG15 (sc-50366, Santa Cruz Biotechnology, Dallas, TX, USA; 1:200), OAS1 (sc-98424, Santa Cruz Biotechnology; 1:400), and IL-6 (Bioss BS-0782R, Life Technologies GmbH, Darmstadt, Germany; 1:500), polyclonal goat anti-IL-10 (sc-1783, Santa Cruz Biotechnology; 1:100) IgG, monoclonal rabbit anti-PKR (ab-32036, Abcam, Cambridge, MA, USA; 1:400) and anti-TMEM119 (ab-209064, Abcam; 1:2000) IgG or monoclonal rat anti-CD45R (RA3-6B2, BD Biosciences, Heidelberg, Germany; 1:2000) and anti-MAC3 (CD107b; AbD Serotec, Oxford, UK; 1:200) IgG overnight at 4 °C. Negative control slides were incubated with rabbit serum (R4505, Merck KGaA, Darmstadt, Germany), goat serum (19140, Sigma-Aldrich Chemie GmbH) or rat serum (MAB006, R&D Systems Inc., Minneapolis, MN, USA). Subsequently, slides were incubated with secondary goat-anti-rabbit (BA-1000, Vector Laboratories, Burlingame, CA, USA; 1:200), rabbit-anti-goat (BA-5000, Vector Laboratories; 1:200), or goat-anti-rat (BA-9401, Vector Laboratories) antibodies for 45 min at room temperature. The avidin–biotin-peroxidase complex (ABC, PK-6100, Vector Laboratories) was used with 3′3-diaminobenzidine (DAB; Merck KGaA) as chromogen. Counterstaining was performed with Mayer’s hematoxylin (Merck KGaA).

TMEV antigen was quantified semiquantitatively in eight areas of the cerebrum (see above) at the level of the hippocampus (0: no positive cell; 1: < 25% positive cells; 2: 25–50% positive cells; 3: > 50% positive cells). The percentage of CD3^+^, CD45R^+^ and Iba-1^+^-positive cells in the perivascular area (Virchow–Robin space) of four vessels present on all three immunostained slides (cut at the level of the hippocampus) was determined. These four vessels were randomly selected, but only vessels with higher numbers of perivascular cells were selected in order to maximize the number of cells evaluated [7]. The number of GFAP^+^ cells, MAC3^+^ cells, and TMEM119^+^ cells within the hippocampus was also evaluated semiquantitatively (0: no increase in cell number; 1: mildly increased cell number; 2: moderately increased cell number; 3: severely increased cell number) [51]. Moreover, the percentage of ISG15, PKR and OAS1 area in the cerebrum at the level of the hippocampus was quantified using analySIS 3.1 software package (SOFT Imaging System, Münster, Germany).

### RNA-seq analysis

The present study re-evaluated data of a previously published RNA-seq analysis, which used the brain samples of 3–5 TMEV- and mock-infected female SJL/JHanHsd and C57BL/6OlaHsd mice euthanized at 4, 7 and 14 dpi [6]. Briefly, RNA was isolated using the RNeasy® Lipid Tissue Mini Kit (Qiagen, Hilden, Germany) following the manufacturers’ instructions and poly A containing mRNA was purified using poly T oligo attached magnetic beads (Illumina, Inc., San Diego, CA, USA). Sequencing was performed on Illumina HiSeq2500 using 50-bp single read. The sequenced libraries were aligned with mouse reference genome (assembly: GRCm38) using splice junction mapper *Tophat2* v1.2.0 [52] with default parameterization. Reads aligned to annotated genes were quantified with *htseq-count* (http://www-huber.embl.de/users/anders/HTSeq) program. Genes involved in IFN-I signaling were selected based on the literature [12, 14, 53–55] and a pairwise comparison of gene expression between mock- and TMEV-infected animals of each strain was performed with *DESeq2* [56]. A log2fold change (FC) of │1.5│ and a *p*-value, adjusted for multiple testing, of ≤ 0.05 were set as cut-offs for significantly differential expression. RNA-seq data can be accessed at GEO/SRA (https://www.ncbi.nlm.nih.gov/geo/) under accession number GSE159226 (https://www.ncbi.nlm.nih.gov/geo/query/acc.cgi?acc=GSE159226).

### RT-qPCR

RNA was transcribed to cDNA using the Omniscript RT Kit (Qiagen), Random Primers (Promega, Mannheim, Germany) and RNase Out (Invitrogen, Darmstadt, Germany). Quantitative PCR was performed for TMEV, *Ifna*, *Ifnb1*, *Ifng*, *Isg15*, *Eif2ak1* (PKR), *Il1b*, *Il4*, *Il6*, *Il10*, *Il12b*, *Tnfa*, *Tgfb1* and three housekeeping genes (*Gapdh*, *Actb*, *Hprt*) using the AriaMx Real-time PCR System (Agilent Technologies Deutschland GmbH), and Brilliant III Ultra-Fast SYBR®QPCR Master Mixes [14, 57–60]. Primers are listed in the supplementary information (Additional file 1: Table S1). Tenfold serial dilution standards ranging from 10^8^ to 10^2^ copies/μL were used for quantification. Results were normalized using a factor calculated from the housekeeping genes [61]. The specificity of each reaction was controlled by melting curve analysis.

### Immunoassay and plaque assay

Samples of frozen cerebral tissue (cut at the level of the hippocampus) were diluted in DMEM to a concentration of 10% and homogenized using Omni Tissue Homogenizer (Süd-Laborbedarf GmbH, Gauting, Germany). A bead-based immunoassay was performed to determine cytokine (IFN-α, IFN-β, IFN-γ, TNF, IL1-β, IL-6, IL-10, IL-12) and chemokine (CCL-2, CCL-5, CXCL-1, CXCL-10) protein expression using a LEGENDplex kit following the manufacturer’s instructions. Cytokine and chemokine concentrations were quantified using the ID7000™ Spectral Cell Analyzer (Sony Biotechnology, San Jose, CA, USA) and data were evaluated with LEGENDplex V8.0 software (Biolegend, San Diego, CA, USA).

The amount of infectious virus in the samples of frozen cerebral tissue of complete and cell-type specific IFNAR-deficient mice was quantified using a plaque assay as described [7].

### Statistics

Data were analyzed using Kruskal–Wallis tests with Dunn’s multiple comparisons tests or Mann–Whitney tests (using the Bonferroni correction for multiple comparisons) with the GraphPad Prism Software Version 8 (Graph-Pad Software, La Jolla, CA, USA). Survival curves were compared using Gehan–Breslow–Wilcoxon tests and the Bonferroni method for the adjustment of *P* values. In addition, Spearman's rank correlation coefficients of PCR and immunoassay data were calculated. A *P* value of < 0.05 was considered as statistically significant.

## Results

### RNA-seq analysis shows strong induction of IFN-I pathway members during acute encephalitis

Data from a previously published RNA-seq analysis were re-evaluated to compare the gene expression of IFN-I pathway members between SJL and C57BL/6 mice at 4, 7 and 14 dpi [6]. For this purpose, fold changes of TMEV-infected vs. mock-infected mice were calculated for each mouse strain. At 4 dpi, 452 and 965 genes were upregulated (fold change > 2) in SJL and C57BL/6 mice, respectively. At 7 dpi, the number of upregulated genes went up to 1029 in SJL mice and down to 719 in C57BL/6. At 14 dpi, only 321 and 139 upregulated genes were detected in SJL and C57BL/6 mice, respectively. Only few downregulated genes (fold change < 0.5) were found at 4 dpi (SJL: 72; C57BL/6: 41), 7 dpi (SJL: 4; C57BL/6: 4) and 14 dpi (SJL: 80; C57BL/6: 2). The gene expression of 107 manually selected genes, including 12 pattern recognition receptors (PRRs), 10 IRFs, 3 IFNs, 4 IFN receptors, 12 signal transducers and 66 ISGs, was analyzed in detail (Table 1; Additional file 1: Table S2) [14, 53].

**Table 1:** Transcriptional changes in the cerebrum of TMEV- and mock-infected SJL and C57BL/6 mice (fold changes)

Gene symbol	Gene title	Strain strain	4 dpi	7 dpi	14 dpi
*Pattern recognition receptors*					
Ddx58 (RIG-I)	DEAD (Asp-Glu-Ala-Asp) box polypeptide 58	SJL	*5.29*	*9.22*	*1.90*
		B6	*6.92*	*7.00*	1.87
Eif2ak2 (PKR)	Eukaryotic translation initiation factor 2-alpha kinase 4	SJL	*6.18*	***11.35***	*2.21*
		B6	*6.36*	*7.99*	*1.96*
Ifih1 (MDA5)	Interferon induced with helicase C domain 1	SJL	*4.28*	*9.25*	*1.94*
		B6	*5.67*	*6.64*	1.80
Tlr3	Toll-like receptor 3	SJL	*2.33*	*3.48*	*1.56*
		B6	*2.82*	*2.72*	1.30
Tlr7	Toll-like receptor 7	SJL	*2.39*	*3.98*	1.54
		B6	*3.35*	*2.94*	1.70
*Interferon regulatory factors*					
Irf1	Interferon regulatory factor 1	SJL	*2.71*	*8.91*	*2.41*
		B6	*2.96*	*5.05*	1.72
Irf3	Interferon regulatory factor 3	SJL	1.11	1.00	1.21
		B6	1.13	1.08	1.12
Irf5	Interferon regulatory factor 5	SJL	*2.07*	*3.12*	1.57
		B6	*2.77*	*2.55*	1.48
Irf7	Interferon regulatory factor 7	SJL	*5.42*	*7.28*	1.78
		B6	*4.77*	*4.87*	1.26
*Type I/II interferons*					
Ifna4	Interferon alpha 4	SJL	*3.70*	1.24	0.89
		B6	1.63	1.09	0.88
Ifnb1	Interferon beta 1	SJL	*4.23*	1.47	0.85
		B6	1.65	1.07	0.94
Ifng	Interferon gamma	SJL	1.56	*5.40*	1.61
		B6	1.53	*2.71*	1.51
*Type I/II interferon receptors*					
Ifnar1	interferon alpha/beta receptor 1	SJL	0.93	*1.21*	0.99
		B6	1.13	1.01	0.93
Ifnar2	interferon alpha/ beta/omega receptor 2	SJL	*1.56*	*1.67*	*1.49*
		B6	*1.54*	1.43	1.09
Ifngr1	Interferon gamma receptor 1	SJL	1.21	*1.84*	1.25
		B6	*1.58*	*1.49*	1.27
Ifngr2	Interferon gamma receptor 2	SJL	1.15	1.12	1.20
		B6	1.00	1.13	1.00
*Signal transducers*					
Irf9	Interferon regulatory factor 9	SJL	*6.06*	*7.33*	*2.75*
		B6	*4.44*	*5.76*	*1.80*
Jak1	Janus kinase 1	SJL	*0.72*	*1.22*	*0.81*
		B6	0.96	1.14	1.11
Socs1	Suppressor of cytokine signaling 1	SJL	*3.67*	*4.84*	1.62
		B6	*2.35*	*3.72*	1.29
Stat1	Signal transducer and activator of transcription 1	SJL	*8.56*	***14.23***	*3.90*
		B6	*5.51*	*9.64*	*2.04*
Stat2	Signal transducer and activator of transcription 2	SJL	*2.53*	*4.99*	1.44
		B6	*3.63*	*4.48*	1.25
Tyk2	Tyrosine kinase 2	SJL	1.15	1.24	1.19
		B6	1.31	1.10	1.02
*Interferon-dependent antiviral effectors*					
Cd74	CD74 antigen	SJL	*2.08*	***15.36***	***10.65***
		B6	*2.79*	*6.51*	*3.97*
Ddx60	DEAD (Asp-Glu-Ala-Asp) box polypeptide 60	SJL	*8.80*	***15.53***	*2.99*
		B6	***10.64***	***10.09***	*2.51*
Ifi44	Interferon-induced protein 44	SJL	*6.91*	***10.78***	*2.48*
		B6	*6.00*	*6.79*	1.34
Ifi202b	Interferon activated gene 202B	SJL	***14.75***	***34.01***	*4.76*
		B6	0.74	0.86	0.67
Ifit1 (Isg56)	Interferon-induced protein with tetratricopeptide repeats 1	SJL	*9.91*	***14.03***	*3.05*
		B6	*7.23*	*9.25*	1.62
Ifit3 (Isg60)	Interferon-induced protein with tetratricopeptide repeats 3	SJL	*9.42*	***10.02***	*2.40*
		B6	*5.95*	*6.35*	1.41
Isg15	ISG15 ubiquitin-like modifier	SJL	*9.27*	*9.32*	*2.38*
		B6	*4.59*	*6.28*	1.06
Mx1	Myxovirus (influenza virus) resistance 1	SJL	*3.96*	*5.63*	1.29
		B6	*5.08*	*4.15*	1.11
Mx2	Myxovirus (influenza virus) resistance 2	SJL	*8.56*	*8.62*	*2.40*
		B6	*5.75*	*5.18*	1.25
Oas1a	2'-5' Oligoadenylate synthetase 1A	SJL	*4.43*	*6.08*	*1.92*
		B6	*4.16*	*4.27*	1.30
Oas1b	2'-5' Oligoadenylate synthetase 1B	SJL	*8.56*	***10.08***	*2.82*
		B6	*5.88*	*5.97*	1.65
Oasl1	2'-5' Oligoadenylate synthetase-like 1	SJL	*2.81*	*3.81*	1.03
		B6	*3.79*	*3.15*	1.08
Oasl2	2'-5' Oligoadenylate synthetase-like 2	SJL	*7.66*	***10.94***	*2.64*
		B6	*6.23*	*8.49*	1.59
Rnasel	Ribonuclease L (2', 5'-oligoisoadenylate synthetase-dependent)	SJL	0.81	*2.08*	0.75
		B6	1.29	1.37	1.14
Rtp4	Receptor transporter protein 4	SJL	***10.01***	***15.45***	*3.66*
		B6	*7.07*	*8.70*	1.91
Trim12a	Tripartite motif-containing 12A	SJL	1.05	1.03	0.77
		B6	*4.79*	*5.09*	*2.31*
Trim12c	Tripartite motif-containing 12C	SJL	1.53	*1.66*	1.18
		B6	*4.61*	*4.68*	*1.96*
Trim30a	Tripartite motif-containing 30A	SJL	*4.42*	***10.37***	*1.92*
		B6	*7.28*	*7.19*	2.06

Significant fold changes are shown in italics letters (adjusted *P* values < 0.05). Bold italic letters indicate significant fold changes > 10. B6: C57BL/6; dpi: days post-infection

In general, changes in gene expression were more pronounced at 4 and 7 dpi than at 14 dpi. Many PRRs (11/12), IRFs (5/10), signal transducers (6/12) and ISGs (45/66) including *Eif2ak2* (PKR), *Isg15* and *Oas1a* were upregulated (fold change > 2) in both mouse strains. The expression of IFN receptors was hardly affected by TMEV infection at the investigated time points in both mouse strains. Interestingly, *Ifna4* was the single IFN-α subtype detected by RNA-seq analysis. *Ifna4* and *Ifnb1* were only upregulated in SJL, but not in C57BL/6 mice at 4 dpi. No significant changes of these two IFN-I were found at 7 and 14 dpi. *Ifng* was upregulated at 7 dpi in both mouse strains but higher in SJL (fold change 5.40) than in C57BL/6 mice (fold change 2.71). *Ifi202b* was the most highly upregulated gene in SJL mice (up to a fold change of 34.01 at 7 dpi), but not affected by TMEV infection in C57BL/6 mice. The expression of *Trim12a* (up to a fold change of 5.09 at 7 dpi) was only increased in C57BL/6 mice. Similarly, *Trim12c* was strongly upregulated in C57BL/6 mice (fold change of 4.68 at 7 dpi) and only mildly induced in SJL mice (fold change of 1.66 at 7 dpi). Transcriptional changes observed in RNA-seq analysis were also validated using RT-qPCR of selected genes. The ratio between the number of *Ifna*, *Ifnb1*, *Irf7*, *Isg15* and *Eif2ak1* mRNA transcripts in SJL and C57BL/6 mice was similar to RNA-seq data (Additional file 1: Fig. S1).

### Minor differences in ISG15, PKR and OAS1 protein expression between SJL and C57BL/6 mice

**Fig. 1:**
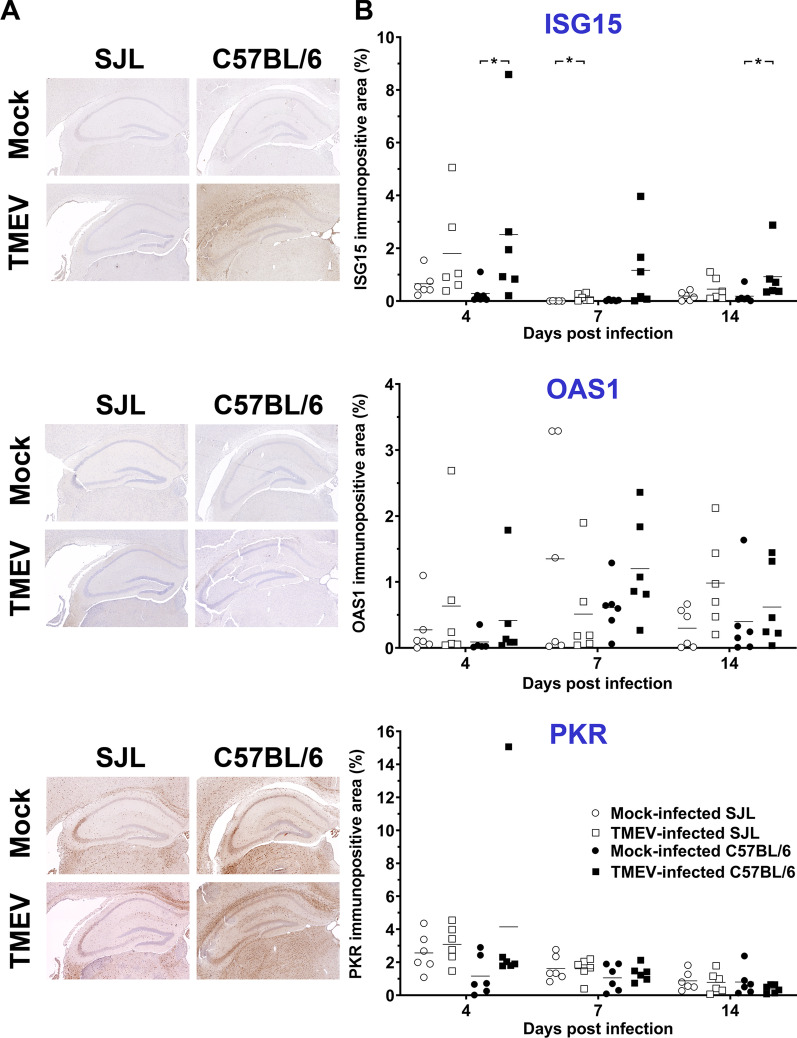
Minor changes in ISG15, OAS1 and PKR protein expression between TMEV- and mock-infected mice. **A** Hippocampus of SJL and C57BL/6 mice at 4 days post-infection (dpi). Immunohistochemistry using the avidin–biotin-peroxidase complex method with the chromogen 3′3-diaminobenzidine and Mayer’s hematoxylin counterstaining. **B** Morphometric analysis of immunohistochemically stained coronal brain sections cut at the level of the hippocampus (1 complete section per mouse evaluated; *n* = 6). Note significantly higher expression of ISG15 in TMEV-infected C57BL/6 mice at 4 and 14 dpi as well as in TMEV-infected SJL mice at 7 dpi compared to the respective mock-infected mice. No significant influence of TMEV infection on OAS1 and PKR protein expression. Kruskal–Wallis tests with Dunn’s multiple comparisons tests: *p < 0.05. Shown are all data points with means. Each data point represents the immunopositive area of one mouse

To compare gene and protein expression of selected ISGs, the percentage of ISG15^+^, PKR^+^ and OAS1^+^ area in the cerebrum of TMEV- and mock-infected SJL and C57BL/6 mice was quantified at 4, 7 and 14 dpi using immunohistochemistry (Fig. 1; Additional file 1: Fig. S2). No statistically significant differences in PKR and OAS1 protein expression were detected between mock- and TMEV-infected mice despite increased mRNA transcripts detected in RNA-seq analysis. In contrast, ISG15 protein expression was induced by TMEV infection in C57BL/6 mice at 4 and 14 dpi and in SJL mice at 7 dpi.

### IFNAR signaling of neuroectodermal cells is essential for survival of TMEV infection

**Fig. 2:**
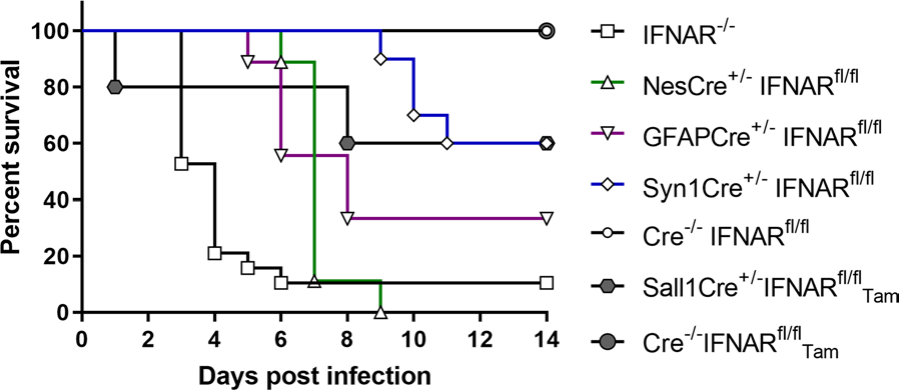
IFNAR signaling of neuroectodermal cells critically affects survival of TMEV-infected mice. Kaplan–Meier survival curves show the survival data of TMEV-infected IFNAR^−/−^ (*n* = 19), NesCre^±^IFNAR^fl/fl^ (*n* = 9), GFAPCre^±^IFNAR^fl/fl^ (*n* = 9), Syn1Cre^±^IFNAR^fl/fl^ (*n* = 10) and Cre^−/−^IFNAR^fl/fl^ mice (*n* = 10) as well as tamoxifen (Tam)-treated Sall1Cre^ER±^ mice (*n* = 5) and Sall1Cre^ER−/−^ control mice (*n* = 5). Note the low survival rate of IFNAR^−/−^ and NesCre^±^IFNAR^fl/fl^ mice, intermediate survival rate of GFAPCre^±^IFNAR^fl/fl^, Syn1Cre^±^IFNAR^fl/fl^ and Sall1Cre^ER±^ mice and 100% survival of Cre^−/−^IFNAR^fl/fl^ and Sall1Cre^ER−/−^ control mice

Complete and cell-type specific IFNAR-deficient mice were used to investigate the role of neurons (Syn1Cre^±^), astrocytes (GFAPCre^±^) and microglia (Sall1Cre^ER±^) in IFNAR signaling during the acute phase of virus-induced encephalitis. While 100% of control Cre^−/−^IFNAR^fl/fl^ mice (*n* = 10) survived until 14 dpi (Fig. 2), only 10.5% of IFNAR^−/−^ mice (*n* = 19; derived from two independent experiments) survived until this time point. 89.5% of IFNAR^−/−^ mice and 100% of NesCre^±^IFNAR^fl/fl^ mice (*n* = 9) reached exclusion criteria at 3–6 dpi and at 6–9 dpi, respectively. 66.7% of GFAPCre^±^IFNAR^fl/fl^ mice (*n* = 9) and 40.0% of Syn1Cre^±^IFNAR^fl/fl^ mice (*n* = 10) had to be euthanized at 5–8 dpi and 9–11 dpi, respectively. Median survival times were 4 dpi for IFNAR^−/−^ mice, 7 dpi for NesCre^±^IFNAR^fl/fl^ mice and 8 dpi for GFAPCre^±^IFNAR^fl/fl^ mice. Gehan–Breslow–Wilcoxon tests showed a significant difference in the survival between IFNAR^−/−^ mice and NesCre^±^IFNAR^fl/fl^, GFAPCre^±^IFNAR^fl/fl^, Syn1Cre^±^IFNAR^fl/fl^ and Cre^−/−^IFNAR^fl/fl^ mice (all *P* values < 0.01), between Cre^−/−^IFNAR^fl/fl^ mice and NesCre^±^IFNAR^fl/fl^ (*P* < 0.01) and GFAPCre^±^IFNAR^fl/fl^ mice (*P* = 0.0275) as well as between NesCre^±^IFNAR^fl/fl^ and Syn1Cre^±^IFNAR^fl/fl^ mice (*P* < 0.01). No statistically significant difference was found between 5 Sall1Cre^ER±^IFNAR^fl/fl^ mice (60% survival rate) and 5 Sall1Cre^ER−/−^IFNAR^fl/fl^ control mice (100% survival rate; *P* = 1.00). In conclusion, cell-type specific knockout mice revealed a major role of IFNAR signaling of neuroectodermal cells and especially of astrocytes for survival after TMEV infection.

### IFNAR signaling of neuroectodermal cells shows no major influence on leukocyte infiltration into the inflamed cerebrum or on the number of astrocytes and microglia in the hippocampus

**Fig. 3:**
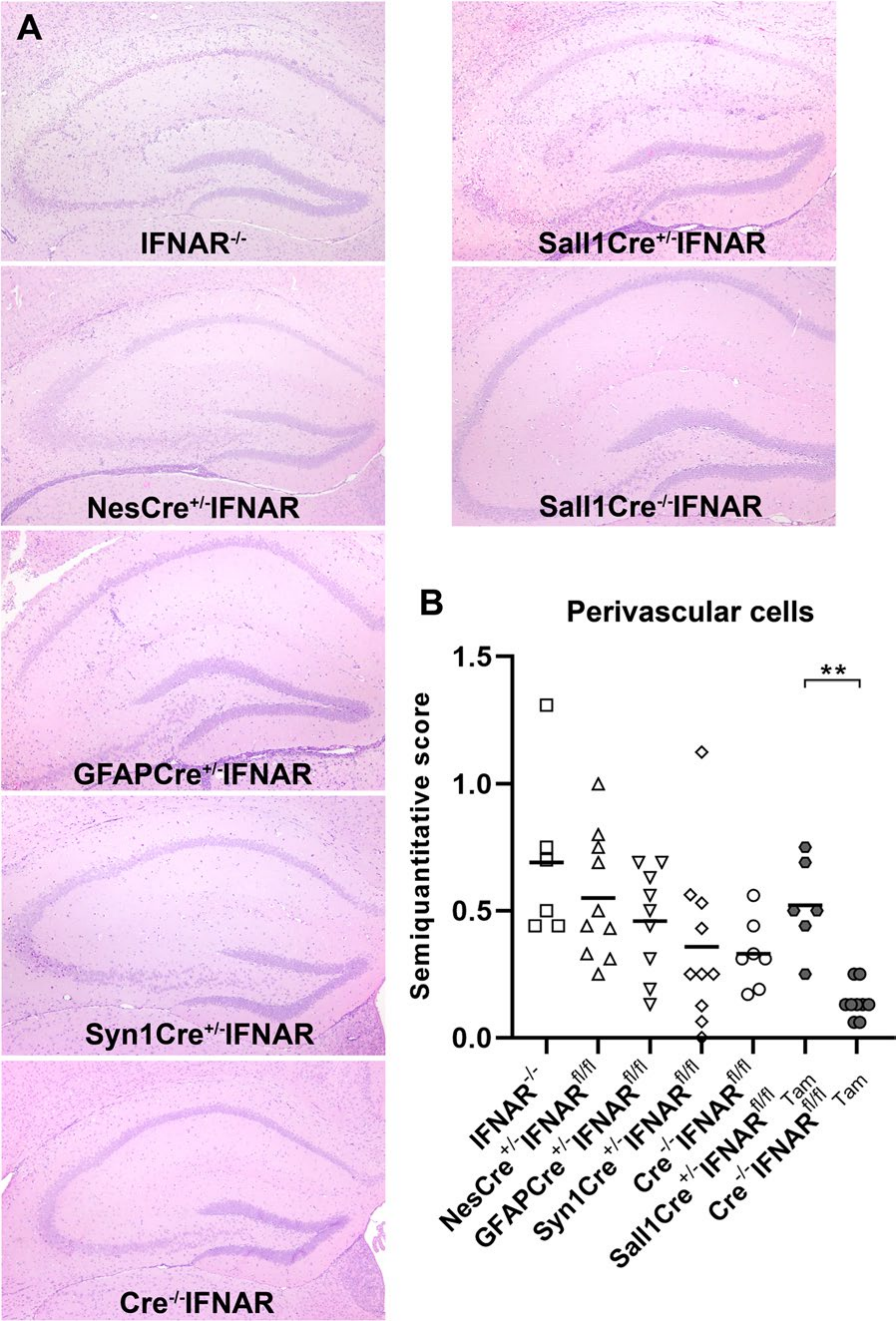
IFNAR signaling of microglia but not of neuroectodermal cells has a significant impact on the number of perivascular mononuclear cells in the cerebrum at 4 days after TMEV-BeAn infection. **A** Histological lesions in the hippocampus of TMEV-infected IFNAR^−/−^, NesCre^±^IFNAR^fl/fl^, GFAPCre^±^IFNAR^fl/fl^, Syn1Cre^±^IFNAR^fl/fl^ and Cre^−/−^IFNAR^fl/fl^ mice as well as tamoxifen (Tam)-treated Sall1Cre^ER±^ mice and Sall1Cre^ER−/−^ control mice. Hematoxylin and eosin staining. **B** Semiquantitative analysis of coronal brain sections cut at the level of the hippocampus (1 complete section per mouse evaluated) revealed a higher number of perivascular mononuclear cells in Tam-treated Sall1CreER^±^ mice (*n* = 6) compared to Sall1CreER^−/−^ control mice (*n* = 9), whereas no significant differences were found between Cre^−/−^IFNAR^fl/fl^ mice (*n* = 7) and IFNAR^−/−^ (*n* = 6), NesCre^±^IFNAR^fl/fl^ (*n* = 10), GFAPCre^±^IFNAR^fl/fl^ (*n* = 9) and Syn1Cre^±^IFNAR^fl/fl^ mice (*n* = 10). Mann–Whitney tests using the Bonferroni correction for multiple comparisons: ** *p* = 0.003. Shown are all data points with means. Each data point represents the semiquantitative score of one mouse

Histological analysis revealed a mild-to-moderate perivascular inflammatory cell infiltration in the cerebrum of all TMEV-infected mice at 4 dpi. Only low numbers of perivascular mononuclear cells were found in the cerebellum and perivascular mononuclear cells were minimal or even absent in the spinal cord at this early time point similar to previous studies [24, 62, 63]. Semiquantitative analysis did not detect significant differences in the number of perivascular mononuclear cells in the cerebrum between Cre^−/−^IFNAR^fl/fl^ mice and IFNAR^−/−^, NesCre^±^IFNAR^fl/fl^, GFAPCre^±^IFNAR^fl/fl^ and Syn1Cre^±^IFNAR^fl/fl^ mice (IFNAR^−/−^ vs. Cre^−/−^IFNAR^fl/fl^ mice: *p* = 0.053). In contrast, Tam-treated Sall1CreER^±^IFNAR^fl/fl^ mice (microglia-specific) showed a higher number of perivascular mononuclear cells into the cerebrum compared to Sall1CreER^−/−^IFNAR^fl/fl^ control mice (Fig. 3). Hippocampal cell loss was generally mild and affected 0–50% of the complete and cell-type specific IFNAR-deficient mice at 4 dpi, but statistically significant differences between these groups were lacking (Additional file 1: Fig. S3).

Immunohistochemistry was used to analyze the composition of the cells in the perivascular area in detail. No significant difference in the percentage of CD3^+^ (T cells), CD45R^+^ (B cells) and Iba-1^+^ (microglia/macrophages) perivascular cells was found between the mouse groups (Additional file 1: Fig. S4). Similarly, immunohistochemistry did not detect significant changes in the number of GFAP^+^, MAC3^+^ cells, and TMEM119^+^ cells in the hippocampus (Additional file 1: Fig. S5). These results indicate that IFNAR signaling of neuroectodermal cells does not have a prominent role in the control of inflammatory cell infiltration into the cerebrum, nor in the number of astrocytes and microglia during TMEV-induced encephalitis at 4 dpi. However, IFNAR signaling of microglia might affect inflammatory cell infiltration in the acute phase of TMEV infection.

### IFNAR signaling of neuroectodermal cells is necessary for the control of virus replication

**Fig. 4:**
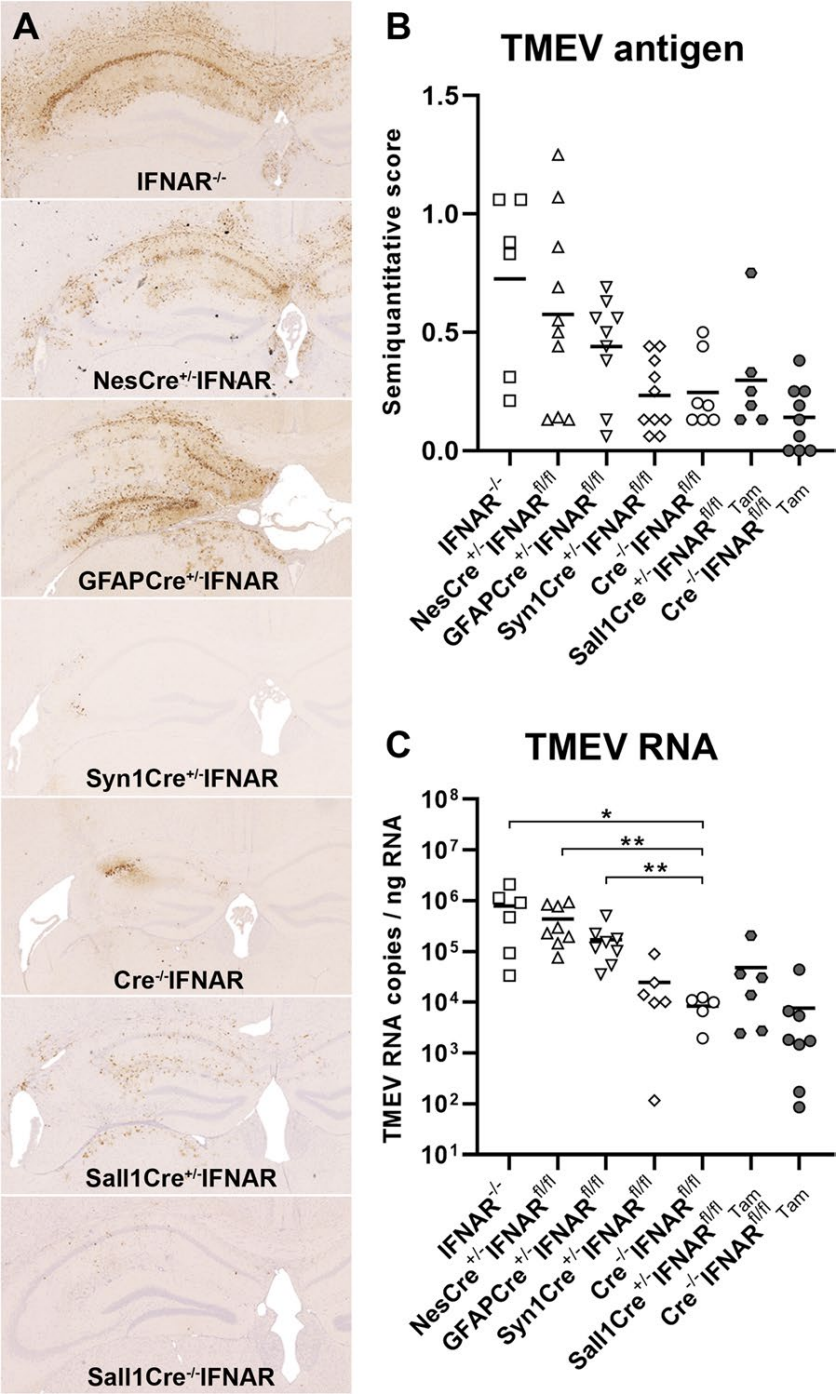
IFNAR signaling of neuroectodermal cells restricts viral load in the cerebrum at 4 days after TMEV-BeAn infection. **A** Viral antigen in the hippocampus of TMEV-infected IFNAR^−/−^, NesCre^±^IFNAR^fl/fl^, GFAPCre^±^IFNAR^fl/fl^, Syn1Cre^±^IFNAR^fl/fl^ and Cre^−/−^IFNAR^fl/fl^ mice as well as tamoxifen (Tam)-treated Sall1Cre^ER±^ mice and Sall1Cre^ER−/−^ control mice. Immunohistochemistry using the avidin–biotin-peroxidase complex method with the chromogen 3′3-diaminobenzidine and Mayer’s hematoxylin counterstaining. **B** Semiquantitative analysis of immunohistochemically stained coronal brain sections cut at the level of the hippocampus (1 complete section per mouse evaluated) did not detect significant differences in the amount of viral antigen between the mouse groups (IFNAR^−/−^: *n* = 6; NesCre^±^IFNAR^fl/fl^: *n* = 10; GFAPCre^±^IFNAR^fl/fl^: *n* = 9; Syn1Cre^±^IFNAR^fl/fl^: *n* = 10; Cre^−/−^IFNAR^fl/fl^: *n* = 7; Sall1CreER^±^: *n* = 6; Sall1CreER^−/−^: *n* = 9). **C** Quantification of TMEV RNA in the cerebrum using RT-qPCR revealed a higher viral load in IFNAR^−/−^ (*n* = 6), NesCre^±^IFNAR^fl/fl^ (*n* = 8) and GFAPCre^±^IFNAR^fl/fl^ mice (*n* = 8) but not Syn1Cre^±^IFNAR^fl/fl^ mice (*n* = 6) compared to Cre^−/−^IFNAR^fl/fl^ mice (*n* = 5). Similar numbers of viral transcripts in tamoxifen (Tam)-treated Sall1Cre^ER±^ mice (*n* = 6) and Sall1Cre^ER−/−^ control mice (*n* = 8). Mann–Whitney tests using the Bonferroni correction for multiple comparisons: * *p* < 0.05; ** *p* < 0.01. Shown are all data points with means. Each data point represents the semiquantitative score (B) or the TMEV RNA copy number (C) of one mouse

Immunohistochemistry revealed large amounts of TMEV antigen in the hippocampus, cerebral cortex and ependymal cells especially in IFNAR^−/−^, NesCre^±^IFNAR^fl/fl^ and GFAPCre^±^IFNAR^fl/fl^ mice at 4 dpi (Fig. 4). Nevertheless, statistical analysis did not detect significant differences in the amount of viral antigen between the mouse groups (IFNAR^−/−^ vs. Cre^−/−^IFNAR^fl/fl^ mice: *p* = 0.067). However, RT-qPCR demonstrated higher TMEV RNA levels in the brain of IFNAR^−/−^, NesCre^±^IFNAR^fl/fl^ and GFAPCre^±^IFNAR^fl/fl^ compared to Cre^−/−^IFNAR^fl/fl^ mice at 4 dpi. No difference in viral load was found between Tam-treated Sall1Cre^ER±^ mice and Sall1Cre^ER−/−^ control mice (Fig. 4). A plaque assay confirmed the presence of infectious virus in the different complete and cell-type specific IFNAR-deficient mice (Additional file 1: Fig. S6). Moreover, a statistical analysis revealed a strong correlation (Spearman’s rank correlation coefficient: *ρ* = 0.7247) between the amount of viral RNA (copy number) and infectious virus (PFU). Consequently, IFNAR signaling of neuroectodermal cells and specifically astrocytes seems to play a major role in controlling virus replication.

### IFNAR deficiency of neuroectodermal cells and microglia affects cytokine and chemokine expression in the cerebrum of TMEV-infected C57BL/6 mice

**Fig. 5:**
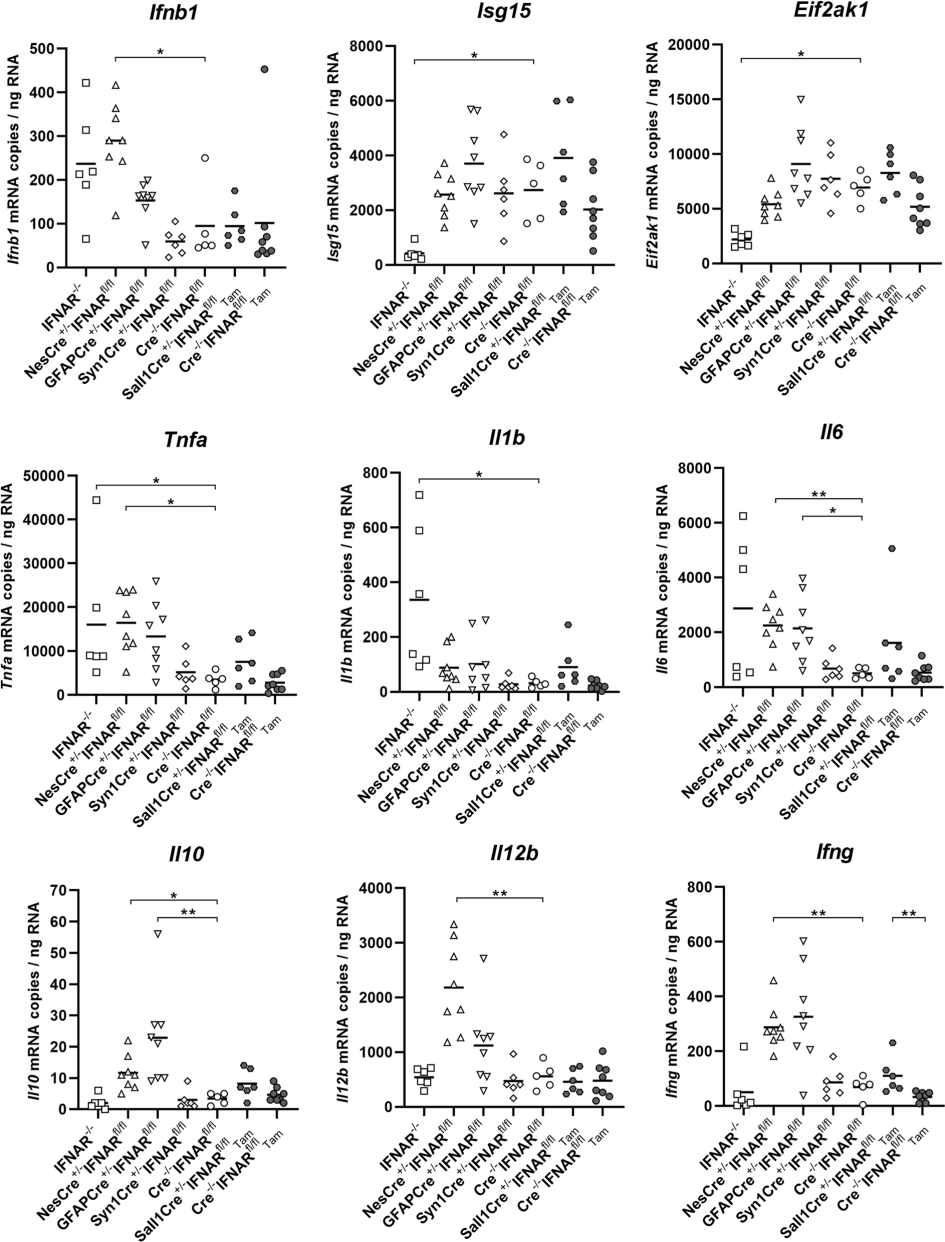
IFNAR deficiency of astrocytes affects *Il6* and *Il10* mRNA transcript levels in the cerebrum at 4 days after TMEV-BeAn infection. RT-qPCR showed a downregulation of *Isg15* and *Eif2ak1* and an upregulation of *Tnfa* and *Il1b* mRNA transcripts in IFNAR^−/−^ mice (*n* = 6) compared to Cre^−/−^IFNAR^fl/fl^ mice (*n* = 5). NesCre^±^IFNAR^fl/fl^ mice (*n* = 8) demonstrated an upregulation of *Ifnb1*, *Tnfa*, *Il6*, *Il10*, *Il12b* and *Ifng* mRNA transcripts compared to Cre^−/−^IFNAR^fl/fl^ mice. *Il6* and *Il10* mRNA transcripts were also higher in GFAPCre^±^IFNAR^fl/fl^ mice (*n* = 8) than in Cre^−/−^IFNAR^fl/fl^ mice, whereas no significant differences were found between Syn1Cre^±^IFNAR^fl/fl^ (*n* = 6) and Cre^−/−^IFNAR^fl/fl^ mice. Only *Ifng* mRNA transcripts were increased in tamoxifen (Tam)-treated Sall1Cre^ER±^ mice (*n* = 6) compared to Sall1Cre^ER−/−^ control mice (*n* = 8). The mRNA copy numbers were normalized using a normalization factor calculated from three housekeeping genes (*Gapdh*, *Actb*, *Hprt*). Mann–Whitney tests using the Bonferroni correction for multiple comparisons: * *p* < 0.05; ** *p* < 0.01. Shown are all data points with means. Each data point represents the mRNA copy number of one mouse

The expression of IFN-I pathway members and several cytokines during TMEV-induced encephalitis was quantified using RT-qPCR. In general, there was a strong correlation (Spearman’s rank correlation coefficient: ρ > 0.6) between the amount of virus and *Ifnb1*, *Tnfa*, *Il1b* and *Il6* mRNA transcripts levels in the investigated mice (Additional file 1: Fig. S7). *Ifnb1*, *Tnfa*, *Il6*, *Il10*, *Il12b* and *Ifng* mRNA transcripts were significantly higher in NesCre^±^IFNAR^fl/fl^ mice than in Cre^−/−^IFNAR^fl/fl^ mice. The number of *Isg15* and *Eif2ak1* (PKR) mRNA transcripts was strongly reduced in IFNAR^−/−^ mice compared to Cre^−/−^IFNAR^fl/fl^ mice confirming the IFN-I dependency of their transcription. In contrast, the number of *Tnfa* and *Il1b* mRNA transcripts was higher in IFNAR^−/−^ than in Cre^−/−^IFNAR^fl/fl^ mice. *Il6* and *Il10* mRNA transcripts were also higher in GFAPCre^±^IFNAR^fl/fl^ mice than in Cre^−/−^IFNAR^fl/fl^ mice, whereas no significant differences were found between Syn1Cre^±^IFNAR^fl/fl^ and Cre^−/−^IFNAR^fl/fl^ mice. Only *Ifng* mRNA transcripts were increased in Tam-treated Sall1Cre^ER±^ mice compared to Sall1Cre^ER−/−^ control mice (Fig. 5).

**Fig. 6:**
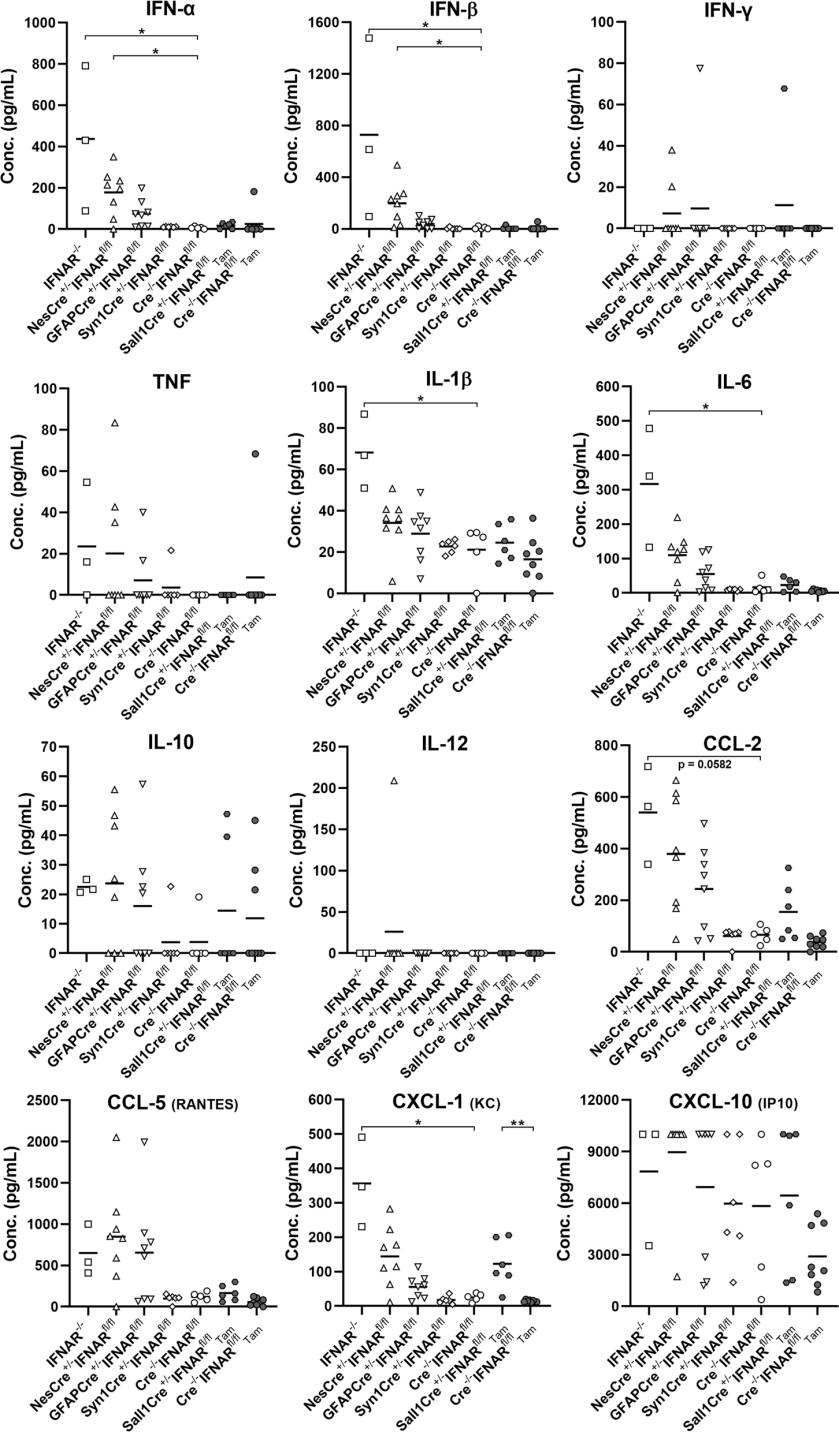
IFNAR deficiency results in increased IFN-α, IFN-β, IL1-β, IL-6, and CXCL-1 protein expression in the cerebrum at 4 days after TMEV-BeAn infection. A bead-based immunoassay demonstrated increased protein levels of IFN-α, IFN-β, IL1-β, IL-6, and CXCL-1 in IFNAR^−/−^ mice (*n* = 3) compared to Cre^−/−^IFNAR^fl/fl^ mice (*n* = 5). IFN-α and IFN-β protein expression was also elevated in NesCre^±^IFNAR^fl/fl^ mice (*n* = 8). Tamoxifen (Tam)-treated Sall1Cre^ER±^ mice (*n* = 6) showed increased CXCL-1 protein levels compared to Sall1Cre^ER−/−^ control mice (*n* = 8). GFAPCre^±^IFNAR^fl/fl^ mice (*n* = 8) and Syn1Cre^±^IFNAR^fl/fl^ (*n* = 6) lacked statistically significant changes in cytokine (IFN-α, IFN-β, IFN-γ, TNF, IL1-β, IL-6, IL-10, IL-12) and chemokine (CCL-2, CCL-5, CXCL-1, CXCL-10) protein expression. Kruskal–Wallis tests with Dunn’s multiple comparisons tests: *p < 0.05; **p < 0.01. Shown are all data points with means. Each data point represents the protein concentration (Conc.) of one mouse

A bead-based immunoassay demonstrated increased protein levels of IFN-α, IFN-β, IL1-β, IL-6, and CXCL-1 in the cerebrum of IFNAR^−/−^ compared to Cre^−/−^IFNAR^fl/fl^ mice at 4 dpi. IFN-α and IFN-β protein expression was also elevated in NesCre^±^IFNAR^fl/fl^ mice, whereas GFAPCre^±^IFNAR^fl/fl^ and Syn1Cre^±^IFNAR^fl/fl^ mice lacked statistically significant changes in cytokine and chemokine protein expression. Tamoxifen (Tam)-treated Sall1Cre^ER±^ mice showed increased CXCL-1 protein levels compared to Sall1Cre^ER−/−^ control mice (Fig. 6). Similar to RT-qPCR data, there was a strong correlation between the viral load (TMEV RNA) and the protein expression of the cytokines IFN-α, IFN-β, IL1-β, and IL-6 as well as the chemokines CCL-2, CCL-5, and CXCL-1 (Additional file 1: Fig. S8).

Immunohistochemistry was used to identify of the cellular origin of IL-6 and IL-10 protein expression in the TMEV-infected brain. IL-6 was mainly found in neurons, whereas perivascular mononuclear cells predominantly lacked IL-6 immunoreactivity. Only few small round and elongated cells as well as medium-sized round cells (most likely representing lymphocytes and microglia/macrophages) showed IL-10 immunoreactivity (Additional file 1: Fig. S9).

These data suggest that increased viral load in IFNAR-deficient mice enhances IFN-α, IFN-β, IL1-β, IL-6, and CXCL-1 expression of TMEV-infected cells particularly neurons. In addition, deficient IFNAR signaling and unrestricted virus replication affects *Il10* and *Ifng* transcript levels.

## Discussion

The present study compared the expression of IFN-I pathway members in the brain of SJL and C57BL/6 mice during the acute phase of TMEV infection. At 4 dpi, roughly two times more genes were upregulated in C57BL/6 than in SJL mice showing a more pronounced reaction to TMEV infection in the resistant mouse strain. *Ifna4* and *Ifnb1* were the only members of the IFN-I gene family detected by RNA-seq analysis. These two IFN-I are constitutively expressed at low levels and their expression can be highly induced in astrocytes and microglia, but not oligodendrocytes, by TLR stimulation [64–66]. Their transcription can be induced without previous protein synthesis, whereas the expression of other IFN-α genes such as *Ifna2*, *5*, *6* and *8* requires previous production of IRF-7 proteins, induced by IFNAR signaling, and their subsequent activation by phosphorylation [67, 68]. The expression of IRF-7 was induced during the acute phase of TMEV infection at least on the transcriptional level. Consequently, the lack of detection of other IFN-α genes might be caused by an inhibition of IRF-7 phosphorylation and/or nuclear translocation, which has been described as a strategy of picornaviruses to suppress IFN-I production [69]. The mRNA of *Ifna1*,* 2*, *5*, *7*,* 9* and *11* was found in the spinal cord during TMEV-IDD using a DNA microarray but the expression of these genes was also not induced by virus infection [14, 70]. In the present study, *Ifna4* and *Ifnb1* were upregulated only in TMEV-infected SJL mice at 4 dpi. Nevertheless, it was previously shown that C57BL/6 mice strongly express IFN-β already at 6 hpi but this expression subsides in the following days [7]. Importantly, IFN-I does not only have antiviral, but also immunosuppressive effects, which can be mediated by increased IL-10 production [71]. Higher IL-10 expression in SJL compared to C57BL/6 mice was already described in the acute phase of TMEV-IDD, which was associated with an increased number of Foxp3^+^ regulatory T cells [72]. Consequently, sustained IFN-β expression in SJL mice might suppress the antiviral immune response in this mouse strain, which eliminates the virus in resistant C57BL/6 mice. Moreover, SJL mice showed a stronger increase in *Ifng* mRNA transcripts than C57BL/6 mice at 7 dpi, which might contribute to a higher MHC-II expression of astrocytes and consecutive activation of potentially autoimmune CD4^+^ T cells in this susceptible mouse strain [64]. In the spinal cord, IFN-γ was highly upregulated between 1 and 7 dpi in TMEV-infected C57BL/6, whereas SJL mice upregulate this type II IFN especially in the chronic phase of TMEV-IDD most likely contributing to T_H_1 cell-mediated immune reactions [63].

The transcription of most ISGs was induced in both mouse strains to a certain extent. Nonetheless, RNA-Seq analysis identified some genes, which were differentially regulated in SJL and C57BL/6 mice. *Ifi202b* was the most highly induced gene in SJL mice but remained unchanged after TMEV infection in C57BL/6 mice. This interferon activated gene belongs to the *Ifi200* family, which includes several murine (*Ifi202a*, *Ifi202b*, *Ifi203*, *Ifi204*, *Mndal*, *Aim2*) and human (*IFI16*, *MNDA*, *IFIX*, *AIM2*) genes [73]. The p202 protein encoded by *Ifi202b* inhibits or stimulates the transcriptional activity of NF-κB in a cell-type specific manner, which modulates innate immune responses and autoimmunity [73, 74]. Nonetheless, the expression of the p202 protein does not exclusively depend on IFNAR signaling [75]. In contrast to *Ifi202b*, the transcription of tripartite motif-containing 12A (*Trim12a*) and *Trim12c* was strongly induced in C57BL/6 but hardly affected by TMEV infection in SJL mice. TRIM12a, TRIM12c and TRIM30a are mouse equivalents of the human TRIM5α protein, which is an E3 ubiquitin ligase and controls NF-κB and IRF-3/7 activity to regulate inflammatory and antiviral immune responses [76, 77]. An in vitro study using 293 T cells demonstrated that TRIM12c boosts innate immunity by activating NF-κB and IFN-I signaling pathways, whereas TRIM12a and TRIM30a are negative regulators of the NF-κB activity. Moreover, TRIM proteins influence T cell development and activation [78]. Consequently, higher expression of specific TRIM proteins in C57BL/6 compared to SJL mice most likely contributes to their resistance to TMEV-IDD. However, further studies are needed to elucidate the impact of increased *Ifi202b* and *Trim12a/c* expression on the innate and adaptive immunity of TMEV-infected SJL and C57BL/6 mice, respectively.

The Mx GTPase pathway, the OAS/RNase L pathway, the PKR pathway and the ISG15 ubiquitin-like pathway are classical IFN-I dependent antiviral effector pathways, which block all steps of viral replication [15]. Nevertheless, most classical inbred mouse strains including SJL and C57BL/6 mice carry *Mx* genes with a large deletion or a nonsense mutation eliminating their antiviral activity [79]. Consequently, the present study focused on the remaining pathways and analyzed the expression of *Oas*, *Pkr*, and *Isg15* genes. The transcription of these genes was highly induced by TMEV infection in SJL and C57BL/6 mice at 4 and 7 dpi, whereas only mild or even no changes were found at 14 dpi. Despite these prominent transcriptional changes, no major influence of TMEV infection was found on the amount of OAS and PKR proteins in the brain of both mouse strains. Several post-transcriptional and translational regulatory processes control protein abundances and explain commonly observed differences between gene and protein expression [80]. Moreover, the OAS/RNase L and PKR pathways are blocked by the TMEV L protein to counteract their antiviral activity [19–23]. Immunohistochemistry detected an increased ISG15 protein expression in TMEV-infected, compared to mock-infected infected mice in SJL mice at 7 dpi and in C57BL/6 mice at 4 and 14 dpi. The ISGylation process affects more than 150 target proteins and various central immune signaling pathways including those mediated by NF-κB, JNK, and IRF-3 [81]. Higher ISG15 protein levels in TMEV-infected mice demonstrate the activation of the IFN-I pathway but the exact role of ISGylation in the pathogenesis of TMEV-IDD has to be analyzed in future studies.

The present study used cell-type specific IFNAR-deficient mice to elucidate the contribution of IFNAR signaling of astrocytes, neurons and microglia to TMEV elimination and survival. The efficiency and specificity of the NesCre^±^IFNAR^fl/fl^ mice has been confirmed in previous publications by using western blot and flow cytometry analysis [82, 83]. Similarly, the specific targeting of astrocytes in the GFAPCre^±^ line and of neurons in the Syn1Cre^±^ line has been demonstrated in different reporter systems [45, 84]. Nonetheless, a cell-type specific sequencing of the Sall1Cre^ER±^ line (used to target microglia) revealed a minor spillover in neuroectodermal cells, which has to be respected in the interpretation of the current knockout experiments [85]. Interestingly, a behavioral study of NesCre mice described an impairment in the acquisition of both contextual- and cued-conditioned fear, although the overall response to stimuli triggering anxiety-like behaviors remained unaltered and alterations in locomotion, general exploratory activity, learning and memory, sociability, startle response and sensorimotor gating were absent in these mice [86]. Moreover, deletion of IFNAR1 in Nes^+^ cells affects the normal cognitive function and synaptic plasticity within the developing CNS [33]. Basal homeostatic interferon signaling in Nes^+^ cells in the brain is also required to prevent Parkinson’s like dementia because it promotes neurite growth and branching, autophagy flux, and α-synuclein degradation in neurons [32]. Consequently, the present NesCre^±^IFNAR^fl/fl^ mice might have changes in their cognitive function, fear learning and behavioral neurobiology. However, the present study investigated the cell-type specific role of IFNAR signaling in the antiviral immune response, which should not be affected by potential neurobehavioral changes.

TMEV-infected IFNAR^−/−^ C57BL/6 mice demonstrated a mean survival of only 3 to 4 days due to unrestricted viral replication. Correspondingly, the mean survival time of cell-type specific IFNAR-deficient mice was inversely related to their viral load in the cerebrum at 4 dpi. TMEV infection of IFNAR^−/−^ 129S2/SP mice resulted in the exclusion of 70% of the animals by 17 dpi, which was also associated with severe encephalitis and high viral loads [36]. At 4 dpi, TMEV antigen can be found predominantly in neurons, astrocytes and ependymal cells, whereas microglia and oligodendroglia are primarily infected at 28 dpi [24, 49]. Higher virus levels in IFNAR^−/−^, NesCre^±^IFNAR^fl/fl^ and GFAPCre^±^IFNAR^fl/fl^ mice but not Syn1Cre^±^IFNAR^fl/fl^ mice compared to Cre^−/−^ mice at 4 dpi demonstrate that IFNAR signaling of neuroectodermal cells and especially of astrocytes plays a dominant role in the restriction of viral replication at this time point. The survival rates of NesCre^±^IFNAR^fl/fl^ (0%), GFAPCre^±^IFNAR^fl/fl^ (33%), and Syn1Cre^±^IFNAR^fl/fl^ mice (60%) underline the dependence of the antiviral immune response of the brain on IFNAR signaling of neuroectodermal cells. A previous study showed that IFN-β treatment confers cultured primary neurons only a low resistance to TMEV infection, which seems to be caused by low basal ISG expression levels of neurons and a low induction of 15 specific ISGs including the apolipoprotein L9 that cooperates with prohibitins to restrict TMEV replication [87, 88]. An inadequate reaction of neurons to IFN-β treatment could explain the limited consequences of IFNAR deficiency on TMEV replication and survival in the present Syn1Cre^±^IFNAR^fl/fl^ mice. Similar to Syn1Cre^±^IFNAR^fl/fl^ mice, no significant differences in viral load and survival rate were found between Sall1Cre^ER±^IFNAR^fl/fl^ mice and their wild-type littermates indicating that IFNAR signaling of microglia does not play a major role in the control of virus replication and survival of TMEV-infected mice as well. However, only five mice of each group were available for the survival experiment limiting its significance. Furthermore, two Sall1Cre^ER±^IFNAR^fl/fl^ mice (40%) had to be euthanized until 8 dpi showing that the knockout of IFNAR-dependent pathways in microglia might at least aggravate TMEV-induced lesions.

Astrocytes infected with TMEV are activated to produce IFN-I, cytokines and chemokines such as IL-1, IL-6, IL-12, TNF, CCL2, CCL-5, CXCL-1, and CXCL10, which trigger and regulate the antiviral immune responses [58, 89–91]. TMEV-infected microglia showed high *Il10* and low *Il12* mRNA levels at 2 dpi, whereas the opposite was the case at 10 dpi indicating a switch from an anti- to a pro-inflammatory phenotype [58]. Few studies investigated the cytokine expression pattern of primary neurons after TMEV infection so far. The present study analyzed the effects of cell-type specific deletion of IFNAR signaling on the expression of downstream targets including ISGs and cytokines using RT-qPCR at 4 dpi. Low *Isg15* and *Eif2ak1* (PKR) mRNA levels in IFNAR^−/−^ mice despite high IFN-β expression confirmed the extensive dependency of their transcription on IFNAR signaling. Nevertheless, IFNAR deletion restricted to astrocytes, neurons or microglia did not change ISG15 and PKR expression measured in the cerebrum, most likely due to the concomitant maintained expression in other CNS-resident cell types.

The mRNA levels of the proinflammatory cytokines *Tnfa*, *Il1*, and *Il6* were highly correlated with viral load, which was most likely mediated by a direct activation of AP-1 and NF-κB transcription factors in astrocytes [58, 63, 91–94]. Likewise, protein levels of IFN-α, IFN-β, IL1-β, IL-6, and CXCL-1 were increased in the present IFNAR^−/−^ mice and highly correlated with viral load. Similar to cytokines, TMEV-induced chemokine gene expression is mediated by TLR-3 signaling and highly dependent on the NF-κB pathway [91, 95]. Immunohistochemistry demonstrated a strong IL-6 expression in neurons, which are also a major target of TMEV infection. Interestingly, IL-6 also represents a major factor in the development of seizures in TMEV-infected C57BL/6 mice [96, 97]. The present results indicate that TMEV infection of neurons directly induces the expression of proinflammatory mediators, which can contribute to ictogenesis. CXCL-1 protein expression was not only elevated in IFNAR^−/−^ mice but also in Sall1Cre^±^IFNAR^fl/fl^ mice, which might be related to higher numbers of perivascular mononuclear cells detected in the brain of these cell-type specific knockout mice. Correspondingly, a recent study revealed that CXCL-1, a chemoattractant for neutrophils and monocytes/macrophages but not T cells, seems to play a critical role in the pathogenesis of TMEV-IDD [90, 98]. NesCre^±^IFNAR^fl/fl^ mice showed increased mRNA levels of *Ifng* and *Il10*, which are largely expressed by T_H_1 cells and regulatory T cells, respectively [99]. The infiltration of different T cell subtypes within the infected CNS is not only dependent on T cell receptor and co-stimulatory signals but also on IFN-I and chemokine secretion mediated by neuronal specific MyD88 signaling [100]. Therefore, increased viral load in the NesCre^±^IFNAR^fl/fl^ mice may trigger stronger MyD88-dependent chemokine responses. Furthermore, a recent study demonstrated that microglia activation is stimulated by neurons and astrocytes by the release of IFNAR-dependent factors, which regulate microglia functions and phenotype and depending on the stage of the infection it may have different pathological outcomes [84, 101]. Nevertheless, the IFNAR-dependent factors orchestrating the complex cross-talk between neurons, astrocytes, and microglia as well as infiltrating immune cells needs to be further delineated during lethal virus-induced encephalitis.

NesCre^±^IFNAR^fl/fl^ mice also showed increased *Il12b* (IL-12 p40) mRNA levels, which might be caused by increased IFN-γ levels, consecutive activation of microglia/macrophages and IRF-1 production [102–106]. Nonetheless, *Ifng* mRNA transcripts were also upregulated in Sall1Cre^±^IFNAR^fl/fl^ mice, which did not show changes in *Il12b* expression. In addition, IFN-γ and IL-12 proteins were hardly detected in the present immunoassay. Interestingly, disparate expression of IL-12 p35 and IL-12 p40 subunits by SJL/J and B10.S macrophages correlate to their resistance to TMEV infection [107]. Moreover, IL-12 p40 is expressed by different myeloid cell subsets in the brain and spinal cord and may play a critical role in the induction of experimental autoimmune encephalitis in C57BL/6 mice [108]. However, the p40 subunit is shared by IL-12 (p35/p40) and IL-23 (p19/p40) and can be secreted as a homodimer (p40/p40) representing an IL-12 receptor antagonist [109]. Most importantly, IL-12 and IL-23 induce T_H_1- and T_H_17-mediated immune responses, which result in TMEV-IDD in SJL mice and TMEV elimination in C57BL/6 mice, respectively [110]. Correspondingly, increased p40 transcription in NesCre^±^IFNAR^fl/fl^ mice might result in a higher production of IL-23 explaining the absence of IL-12 proteins in the brain of TMEV-infected C57BL/6 mice.

In conclusion, the present data indicate that substantial differences in *Ifi202b* and *Trim12a* expression levels between SJL and C57BL/6 mice might influence their susceptibility to TMEV-induced CNS lesions. The restriction of viral replication necessary for survival of infected mice is strongly dependent on IFNAR signaling of neuroectodermal cells especially astrocytes, which thereby also controls the expression of key pro- and anti-inflammatory mediators in the virus-infected brain.

## Supplementary Information

**Additional file 1: Table S1.** Summary of primer pairs used for reverse transcriptase polymerase chain reaction. **Table S2.** Transcriptional changes in the cerebrum of TMEV- and mock-infected SJL and C57BL/6 mice (fold changes). **Fig. S1.** RT-qPCR of the cerebrum at 4, 7 and 14 days after TMEV-BeAn infection (dpi). *lfna* mRNA transcripts were downregulated in TMEV- compared to mock-infected SJL and C57BL/6 mice at 14 dpi, whereas no significant impact of TMEV infection on *Ifnb1* mRNA transcripts was found. *Irf7*, *Isg15* and *Eif2ak1* mRNA transcripts were increased in TMEV- compared to mock-infected SJL and C57BL/6 mice at 4, 7 and 14 dpi except for *Isg15* mRNA transcripts in C57BL/6 mice at 14 dpi. Mock-infected SJL: n=5 (4, 7, 14 dpi); TMEV-infected SJL: n=6 (4,7 dpi), n=5 (14 dpi); mock-infected C57BL/6 mice: n=6 (4, 14 dpi), n=5 (7 dpi); TMEV-infected C57BL/6 mice: n=4 (4 dpi), n=3 (7 dpi), n=5 (14 dpi). The mRNA copy numbers were normalized using a normalization factor calculated from three housekeeping genes (*Gapdh*, *Actb*, *Hprt*). Mann Whitney tests: * p < 0.05; ** p < 0.01. Shown are all data points with means. Each data point represents the mRNA copy number of one mouse. **Fig. S2.** ISG15, OAS1 and PKR protein expression in the brain of TMEV-infected C57BL/6 mice. Shown is the hippocampus of TMEV-infected C57BL/6 mice at 4 (ISG15 and PKR) and 7 (OAS1) days post infection. Immunohistochemistry using the avidin-biotin-peroxidase complex method with the chromogen 3’3-diaminobenzidine and Mayer’s hematoxylin counterstaining. **Fig. S3.** Hippocampal cell loss at 4 days after TMEV-BeAn infection. No significant differences were found between TMEV-infected IFNAR^-/-^ (n=6), NesCre^+/-^IFNAR^fl/fl^ (n=10), GFAPCre^+/-^IFNAR^fl/fl^ (n=9), Syn1Cre^+/-^IFNAR^fl/fl^ (n=10) and Cre^-/-^IFNAR^fl/fl^ mice (n=7) as well as Tam-treated Sall1CreER^+/-^IFNAR^fl/fl^ (n=6) and Sall1CreER^-/-^IFNAR^fl/fl^ mice (n=9). Shown are all data points with means. Each data point represents the semiquantitative score of one mouse. **Fig. S4.** Percentages of perivascular CD3^+^ T cells, CD45R^+^ B cells and Iba-1^+^ macrophages in the cerebrum at 4 days after TMEV-BeAn infection. No significant differences in the percentages of perivascular mononuclear cells were found between TMEV-infected IFNAR^-/-^ (n=6), NesCre^+/-^IFNAR^fl/fl^ (n=8), GFAPCre^+/-^IFNAR^fl/fl^ (n=6), Syn1Cre^+/-^IFNAR^fl/fl^ (n=6) and Cre^-/-^IFNAR^fl/fl^ mice (n=6) as well as Tam-treated Sall1CreER^+/-^IFNAR^fl/fl^ (n=6) and Sall1CreER^-/-^IFNAR^fl/fl^ mice (n=9). Shown are all data points with means. Each data point represents the percentage of immunopositive cells of one mouse. **Fig. S5.** GFAP^+^, MAC3^+^, and TMEM119^+^ cells in the hippocampus at 4 days after TMEV-BeAn infection. No significant differences in the semiquantitative analysis of GFAP^+^, MAC3^+^ and TMEM119^+^ cells were found between TMEV-infected IFNAR^-/-^, NesCre^+/-^IFNAR^fl/fl^, GFAPCre^+/-^IFNAR^fl/fl^, Syn1Cre^+/-^IFNAR^fl/fl^ and Cre^-/-^IFNAR^fl/fl^ mice (n=5-10). Shown are all data points with means. Each data point represents the semiquantitative score of immunopositive cells of one mouse. **Fig. S6.** A plaque assay confirms the presence of infectious virus in the brain at 4 days after TMEV-BeAn infection. Infectious virus was found in TMEV-infected IFNAR^-/-^, NesCre^+/-^IFNAR^fl/fl^, GFAPCre^+/-^IFNAR^fl/fl^, Syn1Cre^+/-^IFNAR^fl/fl^ and Cre^-/-^IFNAR^fl/fl^ mice as well as Tam-treated Sall1CreER^+/-^IFNAR^fl/fl^ and Sall1CreER^-/-^IFNAR^fl/fl^ mice. Shown are all data points with means. Each data point represents the plaque-forming units/ml of one mouse (n=3). **Fig. S7.** Nonparametric Spearman correlation coefficients of RT-qPCR data of the cerebrum at 4 days after TMEV-BeAn infection. **Fig. S8.** Nonparametric Spearman correlation coefficients of TMEV RNA and cytokine array data of the cerebrum at 4 days after TMEV-BeAn infection. **Fig. S9.** TMEV antigen and IL-6 and IL-10 protein expression in the brain of a TMEV-infected NesCre^+/-^IFNAR^fl/fl^ mouse. Shown is the hippocampus of a TMEV-infected NesCre^+/-^IFNAR^fl/fl^ mouse at 4 days post infection (serial sections of the animal shown in Fig. 3). TMEV: Note high amount of TMEV antigen in the hippocampus. IL-6: There is a strong IL-6 expression by hippocampal neurons (upper inset), whereas most perivascular mononuclear cells lack an IL-6 immunoreaction (lower inset). IL-10: IL-10 immunoreactivity was only found in few small round and elongated cells (upper inset) as well as medium-sized round cells (arrow; lower inset). Immunohistochemistry using the avidin-biotin-peroxidase complex method with the chromogen 3’3-diaminobenzidine and Mayer’s hematoxylin counterstaining (serial sections).

## Data Availability

The datasets used and/or analyzed during the current study are available from the corresponding author on reasonable request. The RNA-seq data can be accessed at GEO/SRA (https://www.ncbi.nlm.nih.gov/geo/) under accession number GSE159226 (https://www.ncbi.nlm.nih.gov/geo/query/acc.cgi?acc=GSE159226).

## Abbreviations

B6: C57BL/6
CNS: Central nervous system
dpi: Days post-infection
ds: Double-stranded
eIF2: Eukaryotic translation initiation factor 2A
HE: Hematoxylin and eosin
IFN-I: Interferon type I
IFNAR: Interferon type I receptor
IRF: Interferon regulatory factor
ISG: Interferon stimulated gene
ISG15: IFN-stimulated protein of 15 kDa
KO: Knockout
L: Leader
NF-κB: Nuclear factor of 'kappa-light-chain-enhancer' of activated B-cells
OAS1: 2′-5′-Oligoadenylatsynthetase 1
PKR: Protein kinase RNA-activated
RNA-seq: RNA-based next generation sequencing
TMEV: Theiler’s murine encephalomyelitis virus
TMEV-IDD: Theiler’s murine encephalomyelitis virus-induced demyelinating disease
