# Supplementary material for: IFNAR signaling of neuroectodermal cells is essential for the survival of C57BL/6 mice infected with Theiler’s murine encephalomyelitis virus
Source: J Neuroinflammation. 2023 Mar 5;20:58. doi: 10.1186/s12974-023-02737-6 (PMC9985866; doi:10.1186/s12974-023-02737-6)

Additional Information

IFNAR signaling of neuroectodermal cells is essential for the survival of C57BL/6 mice infected with Theiler's murine encephalomyelitis virus

Melanie Bühler, Dandan Li, Lin Li, Sandra Runft, Inken Waltl, Andreas Pavlou, Ulrich Kalinke, Malgorzata Ciurkiewicz, Jochen Huehn, Stefan Floess, Andreas Beineke, Wolfgang Baumgärtner, Ingo Gerhauser

Table S1: Summary of primer pairs used for reverse transcriptase polymerase chain reaction.

Gene	Acc. no.	Primer direction	Primer position in mRNA	Sequence of primer from 5′→3′	Amplicon length
Actb	NM_007393.5	forward	698-717	GGC TAC AGC TTC ACC ACC AC	233 bp
		reverse	911-930	ATG CCA CAG GAT TCC ATA CC	
***Eif2ak1* (PKR)**	NM_011163.4	forward	1039-1062	GTA CAA GCG CTG GCA GAA CTC AAT	125 bp
		reverse	1143-1163	AAG AGG CAC CGG GTT TTG TAT	
Gapdh	NM_001289726.2	forward	371-390	GAG GCC GGT GCT GAG TAT GT	288 bp
		reverse	639-658	GGT GGC AGT GAT GGC ATG GA	
Hprt	NM_013556.2	forward	646-665	GGA CCT CTC GAA GTG TTG GA	169 bp
		reverse	795-814	TTG CGC TCA TCT TAG GCT TT	
Ifna	various*	forward	various*	ATG GCT AGR CTC TGT GCT TTC CT	512-524 bp*
		reverse		AGG GCT CTC CAG AYT TCT GCT CTG	
Ifnb1	NM_010510.2	forward	206-230	TGA ATG GAA AGA TCA ACC TCA CCT A	76 bp
		reverse	260-281	CTC TTC TGC ATC TTC TCC GTC A	
Ifng	NM_008337.2	forward	175–194	CAC GGC ACA GTC ATT GAA AG	144 bp
		reverse	299–318	AAT CTG GCT CTG CAG GAT TT	
Il1b	NM_008361.4	forward	311-330	AGC TAC CTG TGT CTT TCC CG	150 bp
		reverse	439-460	AGT GCA GTT GTC TAA TGG GAA C	
Il4	NM_021283.2	forward	224-245	CCT CAC AGC AAC GAA GAA CAC C	156 bp
		reverse	358-379	CAT CGA AAA GCC CGA AAG AGT C	
Il6	NM_031168.2	forward	250-269	GTT CTC TGG GAA ATC GTG GA	176 bp
		reverse	404-425	CCA GAG GAA ATT TTC AAT AGG C	
Il10	NM_010548.2	forward	307-326	CCA AGC CTT ATC GGA AAT GA	162 bp
		reverse	449-468	TTT TCA CAG GGG AGA AAT CG	
Il12b	NM_001303244.1	forward	929-948	AGG TGC GTT CCT CGT AGA GA	241 bp
		reverse	1150-1169	AAA GCC AAC CAA GCA GAA GA	
Irf7	NM_016850.3	forward	468-487	CGA GTG CTG TTT GGA GAC TG	156 bp
		reverse	604-623	GGC CTT GAA GAT CTG TGC AT	
Isg15	NM_015783.3	forward	333-351	AAC TGC AGC GAG CCT CTG A	105 bp
		reverse	412-437	CAC CTT CTT CTT AAG CGT GTC TAC AG	
Tgfb1	NM_011577.1	forward	1719–1738	TTG CTT CAG CTC CAC AGA GA	183 bp
		reverse	1882–1901	TGG TTG TAG AGG GCA AGG AC	
**TMEV**	M16020.1	forward	193–214	GAC TAA TCA GAG GAA CGT CAG C	130 bp
		reverse	303–322	GTG AAG AGC GGC AAG TGA GA	
Tnfa	NM_013693.2	forward	257–276	GCC TCT TCT CAT TCC TGC TT	203 bp
		reverse	440–459	CAC TTG GTG GTT TGC TAC GA	

* ***Ifna1*:** NM_010502.2; ***Ifna2*:** NM_010503.2; ***Ifna4*:** NM_010504.2; ***Ifna5*:** NM_010505.2; ***Ifna6*:** NM_206871.1; ***Ifna7*:** NM_008334.3; ***Ifna9*:** NM_010507.1; ***Ifna11*:** NM_008333.2; ***Ifna12*:** NM_177361.2; ***Ifna13*:** NM_177347.2; ***Ifna14*:** NM_206975.1; ***Ifna15*:** NM_206870.1; ***Ifna16*:** NM_206867.1

Table S2: Transcriptional changes in the cerebrum of TMEV- and mock-infected SJL and C57BL/6 mice (fold changes).

Gene Symbol	Gene Title	Strain	4 dpi	7 dpi	14 dpi
**Pattern Recognition Receptors**					
**Ddx58** (RIG-I)	DEAD (Asp-Glu-Ala-Asp) box polypeptide 58	SJL	*5.29*	*9.22*	*1.90*
		B6	*6.92*	*7.00*	1.87
**Eif2ak2** (PKR)	Eukaryotic translation initiation factor 2-alpha kinase 4	SJL	*6.18*	***11.35***	*2.21*
		B6	*6.36*	*7.99*	*1.96*
**Ifih1** (MDA5)	Interferon induced with helicase C domain 1	SJL	*4.28*	*9.25*	*1.94*
		B6	*5.67*	*6.64*	1.80
**Tlr1**	Toll-like receptor 1	SJL	*2.12*	*2.65*	*1.95*
		B6	*3.57*	*2.78*	1.81
**Tlr2**	Toll-like receptor 2	SJL	*2.65*	*5.63*	*2.06*
		B6	*4.89*	*3.85*	1.86
**Tlr3**	Toll-like receptor 3	SJL	*2.33*	*3.48*	*1.56*
		B6	*2.82*	*2.72*	1.30
**Tlr4**	Toll-like receptor 4	SJL	1.44	*3.52*	1.15
		B6	*2.75*	*3.09*	1.84
**Tlr5**	Toll-like receptor 5	SJL	0.96	1.10	0.78
		B6	1.51	0.96	0.95
**Tlr7**	Toll-like receptor 7	SJL	*2.39*	*3.98*	1.54
		B6	*3.35*	*2.94*	1.70
**Tlr8**	Toll-like receptor 8	SJL	0.93	*4.30*	*2.17*
		B6	*3.63*	*2.43*	1.34
**Tlr9**	Toll-like receptor 9	SJL	*5.98*	*5.98*	0.90
		B6	*5.11*	*3.86*	*2.20*
**Tlr13**	Toll-like receptor 13	SJL	1.35	*4.13*	1.18
		B6	*3.66*	*2.61*	1.73
**Interferon Regulatory Factors**					
**Irf1**	Interferon regulatory factor 1	SJL	*2.71*	*8.91*	*2.41*
		B6	*2.96*	*5.05*	1.72
**Irf2**	Interferon regulatory factor 2	SJL	1.11	*1.53*	1.16
		B6	*1.30*	*1.52*	1.14
**Irf3**	Interferon regulatory factor 3	SJL	1.11	1.00	1.21
		B6	1.13	1.08	1.12
**Irf4**	Interferon regulatory factor 4	SJL	1.37	*3.93*	*2.03*
		B6	1.31	*2.24*	*2.22*
**Irf5**	Interferon regulatory factor 5	SJL	*2.07*	*3.12*	1.57
		B6	*2.77*	*2.55*	1.48
**Irf6**	Interferon regulatory factor 6	SJL	0.80	0.88	0.85
		B6	1.07	1.28	1.13
**Irf7**	Interferon regulatory factor 7	SJL	*5.42*	*7.28*	1.78
		B6	*4.77*	*4.87*	1.26
**Irf8**	Interferon regulatory factor 8	SJL	*2.29*	*5.52*	*2.17*
		B6	*3.10*	*3.58*	1.80
**Nfkb1** (p105)	Nuclear factor kappa B subunit 1	SJL	1.10	*1.78*	0.95
		B6	*1.67*	*1.47*	1.13
**Rela** (p65)	v-rel reticuloendotheliosis viral oncogene homolog A	SJL	1.17	1.28	1.17
		B6	1.21	1.14	1.10

Table S2 (*Continued*)

Gene Symbol	Gene Title	Strain	4 dpi	7 dpi	14 dpi
**Type I/II Interferons**					
**Ifna4**	**Interferon alpha 4**	**SJL**	*3.70*	1.24	0.89
		**B6**	1.63	1.09	0.88
**Ifnb1**	**Interferon beta 1**	**SJL**	*4.23*	1.47	0.85
		**B6**	1.65	1.07	0.94
**Ifng**	**Interferon gamma**	**SJL**	1.56	*5.40*	1.61
		**B6**	1.53	*2.71*	1.51
**Type I/II Interferon Receptors**					
**Ifnar1**	**interferon alpha/beta receptor 1**	**SJL**	0.93	*1.21*	0.99
		**B6**	1.13	1.01	0.93
**Ifnar2**	**interferon alpha/ beta/omega receptor 2**	**SJL**	*1.56*	*1.67*	*1.49*
		**B6**	*1.54*	1.43	1.09
**Ifngr1**	**Interferon gamma receptor 1**	**SJL**	1.21	*1.84*	1.25
		**B6**	*1.58*	*1.49*	1.27
**Ifngr2**	**Interferon gamma receptor 2**	**SJL**	1.15	1.12	1.20
		**B6**	1.00	1.13	1.00
**Signal Transducers**					
**Irf9**	**Interferon regulatory factor 9**	**SJL**	*6.06*	*7.33*	*2.75*
		**B6**	*4.44*	*5.76*	*1.80*
**Jak1**	**Janus kinase 1**	**SJL**	*0.72*	*1.22*	*0.81*
		**B6**	0.96	1.14	1.11
**Socs1**	**Suppressor of cytokine signaling 1**	**SJL**	*3.67*	*4.84*	1.62
		**B6**	*2.35*	*3.72*	1.29
**Socs2**	**Suppressor of cytokine signaling 2**	**SJL**	1.15	0.99	1.08
		**B6**	0.96	0.93	0.92
**Socs3**	**Suppressor of cytokine signaling 3**	**SJL**	*2.75*	*4.15*	1.59
		**B6**	*2.26*	*2.02*	1.05
**Stat1**	**Signal transducer and activator of transcription 1**	**SJL**	*8.56*	***14.23***	*3.90*
		**B6**	*5.51*	*9.64*	*2.04*
**Stat2**	**Signal transducer and activator of transcription 2**	**SJL**	*2.53*	*4.99*	1.44
		**B6**	*3.63*	*4.48*	1.25
**Stat3**	**Signal transducer and activator of transcription 3**	**SJL**	0.99	*2.39*	1.06
		**B6**	*1.91*	*2.03*	1.29
**Stat5a**	**Signal transducer and activator of transcription 5A**	**SJL**	1.62	*2.35*	1.27
		**B6**	*1.82*	1.62	1.61
**Stat5b**	**Signal transducer and activator of transcription 5B**	**SJL**	1.00	1.08	1.01
		**B6**	1.08	1.11	1.61
**Stat6**	**Signal transducer and activator of transcription 6**	**SJL**	1.13	*2.01*	1.27
		**B6**	*1.75*	*1.59*	1.34
**Tyk2**	**Tyrosine kinase 2**	**SJL**	1.15	1.24	1.19
		**B6**	1.31	1.10	1.02
**Interferon-Dependent Antiviral Effectors**					
**Adar**	**Adenosine deaminase. RNA-specific**	**SJL**	*0.69*	1.31	*0.64*
		**B6**	1.19	1.30	1.07
**Apobec3**	**Apolipoprotein B mRNA editing enzyme. catalytic polypeptide 3**	**SJL**	*2.69*	*3.34*	1.77
		**B6**	*3.72*	*4.57*	1.47
**Apol9a**	**Apolipoprotein L 9a**	**SJL**	*2.91*	*2.42*	1.33
		**B6**	*2.24*	*2.05*	1.05

Table S2 (*Continued*)

Gene Symbol	Gene Title	Strain	4 dpi	7 dpi	14 dpi
**Apol9b**	**Apolipoprotein L 9b**	**SJL**	*3.54*	*3.93*	1.75
		**B6**	*3.82*	*3.45*	1.30
**Bst2 (Tetherin)**	**Bone marrow stromal cell antigen 2**	**SJL**	*7.30*	*8.86*	*2.76*
		**B6**	*4.42*	*5.01*	1.29
**Mb21d1 (C6orf150)**	**Mab-21 domain containing 1**	**SJL**	*1.65*	*2.99*	1.14
		**B6**	*2.01*	*2.21*	1.25
**Cd74**	**CD74 antigen**	**SJL**	*2.08*	***15.36***	***10.65***
		**B6**	*2.79*	*6.51*	*3.97*
**Ch25h**	**Cholesterol 25-hydroxylase**	**SJL**	*3.64*	*3.71*	1.44
		**B6**	*3.28*	*2.17*	1.13
**Ddit4**	**DNA-damage-inducible transcript 4**	**SJL**	1.08	0.78	1.06
		**B6**	0.99	0.85	0.92
**Ddx17**	**DEAD (Asp-Glu-Ala-Asp) box polypeptide 17**	**SJL**	*0.81*	0.95	*0.76*
		**B6**	1.16	0.98	1.00
**Ddx60**	**DEAD (Asp-Glu-Ala-Asp) box polypeptide 60**	**SJL**	*8.80*	***15.53***	*2.99*
		**B6**	***10.64***	***10.09***	*2.51*
**Gbp2**	**Guanylate binding protein 2**	**SJL**	*3.11*	*8.55*	*2.21*
		**B6**	*4.68*	*8.10*	1.96
**Hpse**	**Heparanase**	**SJL**	1.52	*4.69*	1.46
		**B6**	1.60	*2.00*	1.41
**Ifi30**	**Interferon gamma inducible protein 30**	**SJL**	*2.34*	*4.12*	*1.94*
		**B6**	*4.04*	*4.23*	1.69
**Ifi35**	**Interferon-induced protein 35**	**SJL**	*4.38*	*5.48*	*2.36*
		**B6**	*3.50*	*4.08*	1.44
**Ifi44**	**Interferon-induced protein 44**	**SJL**	*6.91*	***10.78***	*2.48*
		**B6**	*6.00*	*6.79*	1.34
**Ifi47**	**Interferon gamma inducible protein 47**	**SJL**	*2.68*	*6.88*	*1.89*
		**B6**	*3.26*	*4.52*	1.41
**Ifi202b**	**Interferon activated gene 202B**	**SJL**	***14.75***	***34.01***	*4.76*
		**B6**	0.74	0.86	0.67
**Ifi203**	**Interferon activated gene 203**	**SJL**	*3.21*	*7.43*	*1.98*
		**B6**	*6.13*	*5.27*	2.03
**Ifit1 (Isg56)**	**Interferon-induced protein with tetratricopeptide repeats 1**	**SJL**	*9.91*	***14.03***	*3.05*
		**B6**	*7.23*	*9.25*	1.62
**Ifit2 (Isg54)**	**Interferon-induced protein with tetratricopeptide repeats 2**	**SJL**	*3.96*	*8.31*	1.59
		**B6**	*5.39*	*6.45*	1.65
**Ifit3 (Isg60)**	**Interferon-induced protein with tetratricopeptide repeats 3**	**SJL**	*9.42*	***10.02***	*2.40*
		**B6**	*5.95*	*6.35*	1.41
**Ifitm1**	**Interferon induced transmembrane protein 1**	**SJL**	1.47	*3.12*	1.32
		**B6**	*1.98*	*2.34*	1.11
**Ifitm2**	**Interferon induced transmembrane protein 2**	**SJL**	*1.96*	*2.27*	*1.93*
		**B6**	*2.06*	*1.78*	1.20
**Ifitm3**	**Interferon induced transmembrane protein 3**	**SJL**	*5.76*	*7.28*	*2.56*
		**B6**	*4.47*	*4.36*	1.21
**Ifitm6**	**Interferon induced transmembrane protein 6**	**SJL**	1.75	*2.02*	1.19
		**B6**	*1.91*	1.90	0.99
**Ifitm7**	**Interferon induced transmembrane protein 7**	**SJL**	1.21	1.18	0.75
		**B6**	*2.05*	0.93	0.92

Table S2 (*Continued*)

Gene Symbol	Gene Title	Strain	4 dpi	7 dpi	14 dpi
**Ifitm10**	**Interferon induced transmembrane protein 10**	**SJL**	*1.28*	0.97	*1.46*
		**B6**	*0.78*	0.80	0.88
**Isg15**	**ISG15 ubiquitin-like modifier**	**SJL**	*9.27*	*9.32*	*2.38*
		**B6**	*4.59*	*6.28*	1.06
**Isg20**	**Interferon-stimulated protein**	**SJL**	*3.62*	*4.64*	*1.88*
		**B6**	*2.79*	*3.50*	1.35
**Map3k14 (Nik)**	**Mitogen-activated protein kinase kinase kinase 14**	**SJL**	1.16	*1.58*	1.05
		**B6**	*1.70*	1.52	1.23
**Mov10**	**Moloney leukemia virus 10**	**SJL**	1.45	1.39	0.95
		**B6**	*1.91*	1.60	1.00
**Ms4a4a**	**Membrane-spanning 4-domains. subfamily A. member 4A**	**SJL**	1.23	1.74	0.80
		**B6**	*2.43*	1.59	1.18
**Mx1**	**Myxovirus (influenza virus) resistance 1**	**SJL**	*3.96*	*5.63*	1.29
		**B6**	*5.08*	*4.15*	1.11
**Mx2**	**Myxovirus (influenza virus) resistance 2**	**SJL**	*8.56*	*8.62*	*2.40*
		**B6**	*5.75*	*5.18*	1.25
**Nampt (Pbef1)**	**Nicotinamide phosphoribosyltransferase**	**SJL**	1.21	*2.03*	1.07
		**B6**	*1.43*	*1.83*	1.10
**Nt5c3**	**5'-nucleotidase. cytosolic III**	**SJL**	*1.36*	1.15	1.40
		**B6**	0.93	1.10	0.96
**Nt5c3b**	**5'-nucleotidase. cytosolic IIIB**	**SJL**	*1.19*	0.92	1.04
		**B6**	0.99	0.88	0.96
**Oas1a**	**2'-5' oligoadenylate synthetase 1A**	**SJL**	*4.43*	*6.08*	*1.92*
		**B6**	*4.16*	*4.27*	1.30
**Oas1b**	**2'-5' oligoadenylate synthetase 1B**	**SJL**	*8.56*	***10.08***	*2.82*
		**B6**	*5.88*	*5.97*	1.65
**Oas1c**	**2'-5' oligoadenylate synthetase 1C**	**SJL**	*2.63*	*2.81*	*2.21*
		**B6**	*2.18*	*1.85*	1.67
**Oas1g**	**2'-5' oligoadenylate synthetase 1G**	**SJL**	*3.75*	*5.06*	1.66
		**B6**	*4.72*	*3.85*	1.33
**Oas2**	**2'-5' oligoadenylate synthetase 2**	**SJL**	*3.87*	*6.27*	*2.30*
		**B6**	*5.81*	*4.79*	1.67
**Oas3**	**2'-5' oligoadenylate synthetase 3**	**SJL**	*3.36*	*7.59*	1.33
		**B6**	*5.39*	*5.49*	1.25
**Oasl1**	**2'-5' oligoadenylate synthetase-like 1**	**SJL**	*2.81*	*3.81*	1.03
		**B6**	*3.79*	*3.15*	1.08
**Oasl2**	**2'-5' oligoadenylate synthetase-like 2**	**SJL**	*7.66*	***10.94***	*2.64*
		**B6**	*6.23*	*8.49*	1.59
**P2ry6**	**Pyrimidinergic receptor P2Y. G-protein coupled. 6**	**SJL**	*1.91*	*2.79*	*1.84*
		**B6**	*2.04*	*2.03*	1.30
**Pml (Trim19)**	**Promyelocytic leukemia**	**SJL**	1.29	*2.61*	0.96
		**B6**	*2.03*	*2.29*	1.13
**Rnasel**	**Ribonuclease L (2'. 5'-oligoisoadenylate synthetase-dependent)**	**SJL**	0.81	*2.08*	0.75
		**B6**	1.29	1.37	1.14
**Rsad2 (Viperin)**	**Radical S-adenosyl methionine domain containing 2**	**SJL**	*3.99*	*6.26*	1.28
		**B6**	*7.76*	*6.06*	1.61
**Rtp4**	**Receptor transporter protein 4**	**SJL**	***10.01***	***15.45***	*3.66*
		**B6**	*7.07*	*8.70*	1.91

Table S2 (*Continued*)

Gene Symbol	Gene Title	Strain	4 dpi	7 dpi	14 dpi
**Samhd1**	**SAM domain and HD domain. 1**	**SJL**	*1.63*	*3.89*	1.23
		**B6**	*2.85*	*3.41*	1.43
**Slc15a3**	**Solute carrier family 15, member 3**	**SJL**	*2.35*	*5.30*	1.63
		**B6**	*3.17*	*2.71*	1.32
**Slc25a28**	**Solute carrier family 25, member 28**	**SJL**	1.22	1.00	1.20
		**B6**	0.90	0.88	0.92
**Ssbp3**	**Single-stranded DNA binding protein 3**	**SJL**	1.04	1.03	*1.24*
		**B6**	0.87	0.89	1.00
**Trex1** (Atrip)	**Three prime repair exonuclease 1 (ATR interacting protein)**	**SJL**	1.12	0.69	0.85
		**B6**	1.16	0.73	0.75
**Trim12a**	**Tripartite motif-containing 12A**	**SJL**	1.05	1.03	0.77
		**B6**	*4.79*	*5.09*	*2.31*
**Trim12c**	**Tripartite motif-containing 12C**	**SJL**	1.53	*1.66*	1.18
		**B6**	*4.61*	*4.68*	*1.96*
**Trim30a**	**Tripartite motif-containing 30A**	**SJL**	*4.42*	***10.37***	*1.92*
		**B6**	*7.28*	*7.19*	2.06
**Ifi209**	**Interferon activated gene 209**	**SJL**	*2.96*	*7.84*	1.62
		**B6**	*8.10*	*8.71*	2.29
**Ifi213**	**Interferon activated gene 213**	**SJL**	1.71	*2.86*	0.97
		**B6**	*8.99*	*7.60*	2.30
**Trim5**	**Tripartite motif-containing 5**	**SJL**	*2.69*	*3.05*	*2.15*
		**B6**	*2.55*	*1.90*	1.30
**Trim25**	**Tripartite motif-containing 25**	**SJL**	*2.59*	*4.69*	1.50
		**B6**	*4.29*	*3.99*	1.67
**Sun2** (Unc84b)	**Sad1 and UNC84 domain containing 2**	**SJL**	0.83	0.97	0.82
		**B6**	0.84	1.07	0.99
**Zbtb16** (Plzf)	**Zinc finger and BTB domain containing 16**	**SJL**	*0.52*	1.11	0.67
		**B6**	1.15	1.23	1.10
**Zc3hav1** (Zap)	**Zinc finger CCCH type, antiviral 1**	**SJL**	*4.15*	*6.77*	*3.22*
		**B6**	*3.86*	*4.12*	1.96
**Cellular Markers**					
**GFAP**	**Glial fibrillary acidic protein**	**SJL**	*2.47*	*3.42*	*1.90*
		**B6**	*3.88*	*3.55*	2.03
**Sall1**	**Sal-like 1**	**SJL**	*1.29*	*1.39*	*1.37*
		**B6**	*1.45*	*1.35*	1.24
**Syp**	**Synaptophysin**	**SJL**	1.01	0.92	1.15
		**B6**	*0.72*	0.82	0.92

Significant fold changes are shown in italics letters (adjusted *P* values < 0.05). Bold letters indicate significant fold changes > 10. B6: C57BL/6; dpi: days post infection.

Fig. S1: RT-qPCR of the cerebrum at 4, 7 and 14 days after TMEV-BeAn infection (dpi). *Ifna* mRNA transcripts were downregulated in TMEV- compared to mock-infected SJL and C57BL/6 mice at 14 dpi, whereas no significant impact of TMEV infection on *Ifnb1* mRNA transcripts was found. *Irf7*, *Isg15* and *Eif2ak1* mRNA transcripts were increased in TMEV-compared to mock-infected SJL and C57BL/6 mice at 4, 7 and 14 dpi except for *Isg15* mRNA transcripts in C57BL/6 mice at 14 dpi. Mock-infected SJL: n=5 (4, 7, 14 dpi); TMEV-infected SJL: n=6 (4,7 dpi), n=5 (14 dpi); mock-infected C57BL/6 mice: n=6 (4, 14 dpi), n=5 (7 dpi); TMEV-infected C57BL/6 mice: n=4 (4 dpi), n=3 (7 dpi), n=5 (14 dpi). The mRNA copy numbers were normalized using a normalization factor calculated from three housekeeping genes (*Gapdh*, *Actb*, *Hprt*). Mann Whitney tests: * p < 0.05; ** p < 0.01. Shown are all data points with means. Each data point represents the mRNA copy number of one mouse.

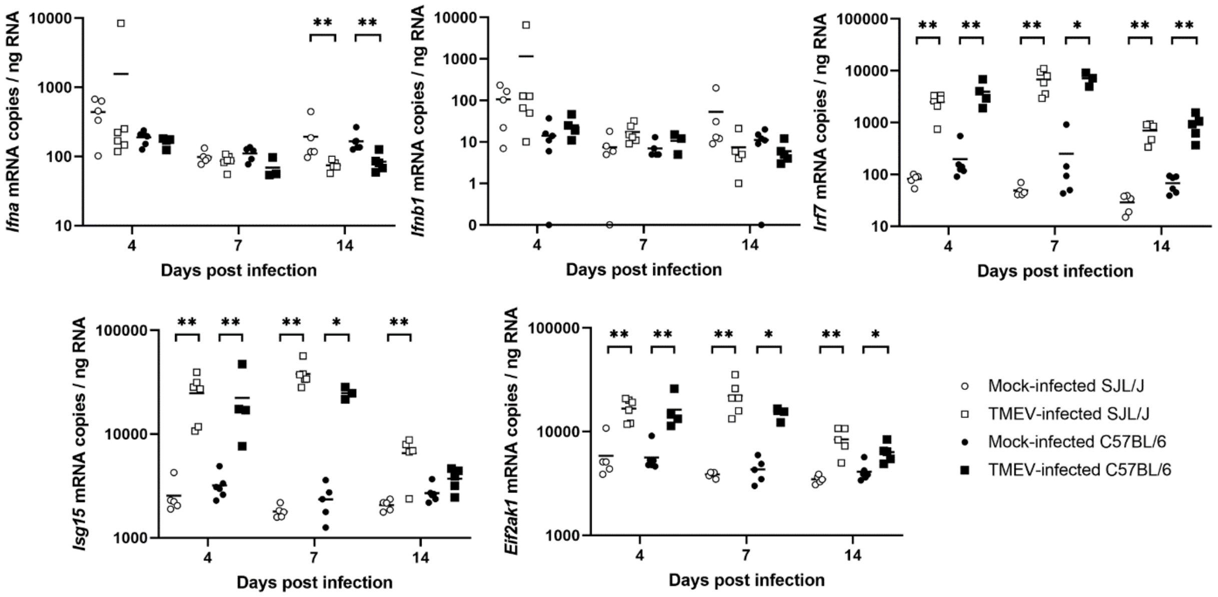

Fig. S2: ISG15, OAS1 and PKR protein expression in the brain of TMEV-infected C57BL/6 mice. Shown is the hippocampus of TMEV-infected C57BL/6 mice at 4 (ISG15 and PKR) and 7 (OAS1) days post infection. Immunohistochemistry using the avidin-biotin-peroxidase complex method with the chromogen 3'3-diaminobenzidine and Mayer's hematoxylin counterstaining.

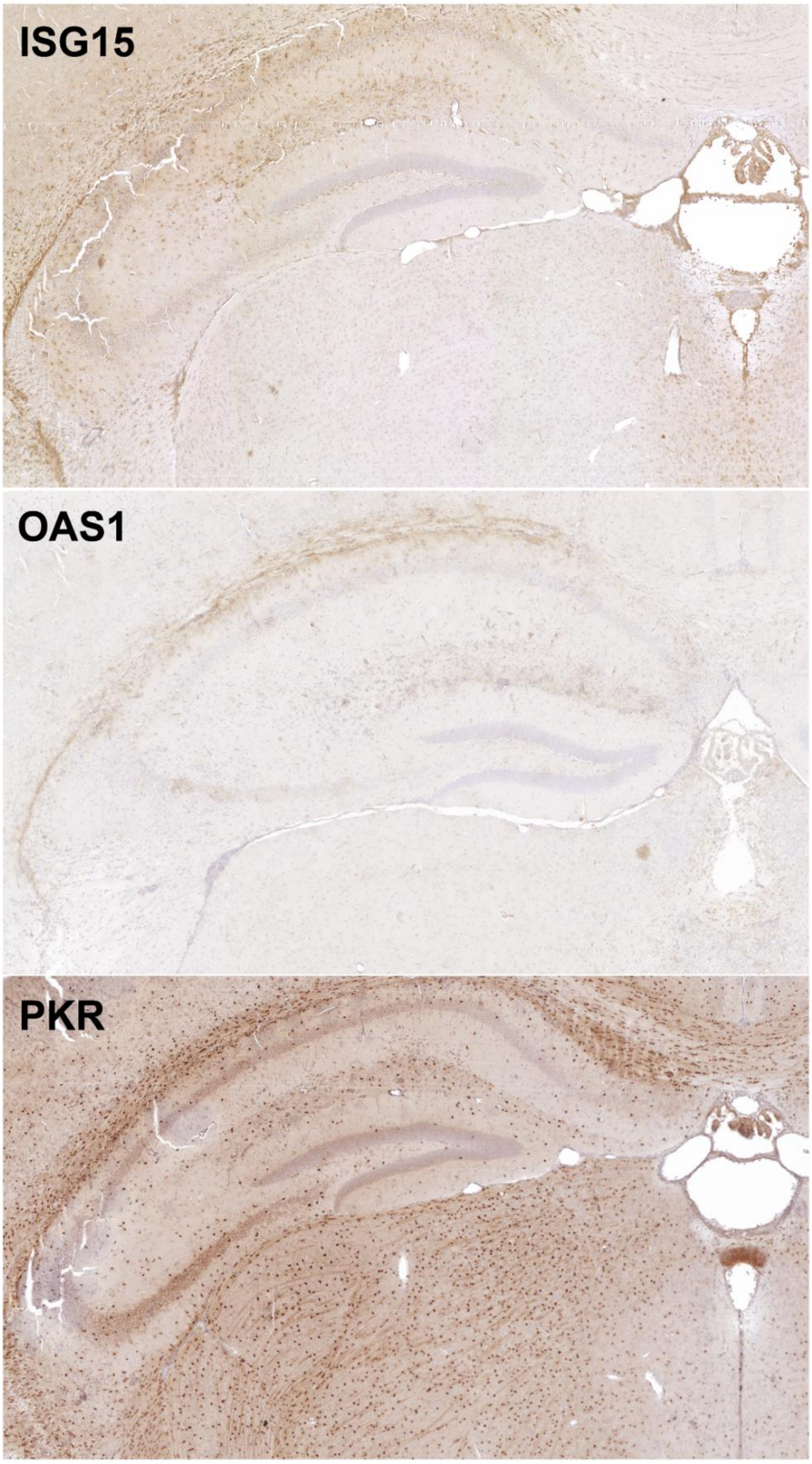

Fig. S3: Hippocampal cell loss at 4 days after TMEV-BeAn infection. No significant differences were found between TMEV-infected IFNAR$^{-/-}$ (n=6), NesCre$^{+/-}$IFNAR$^{fl/fl}$ (n=10), GFAPCre$^{+/-}$IFNAR$^{fl/fl}$ (n=9), Syn1Cre$^{+/-}$IFNAR$^{fl/fl}$ (n=10) and Cre$^{-/-}$IFNAR$^{fl/fl}$ mice (n=7) as well as Tam-treated Sall1CreER$^{+/-}$IFNAR$^{fl/fl}$ (n=6) and Sall1CreER$^{-/-}$IFNAR$^{fl/fl}$ mice (n=9). Shown are all data points with means. Each data point represents the semiquantitative score of one mouse.

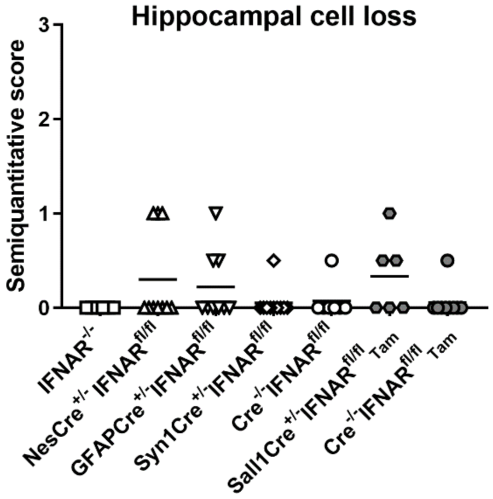

Fig. S4: Percentages of perivascular CD3^{+} T cells, CD45R^{+} B cells and Iba-1^{+} macrophages in the cerebrum at 4 days after TMEV-BeAn infection. No significant differences in the percentages of perivascular mononuclear cells were found between TMEV-infected IFNAR$^{-/-}$ (n=6), NesCre$^{+/-}$IFNAR$^{fl/fl}$ (n=8), GFAPCre$^{+/-}$IFNAR$^{fl/fl}$ (n=6), Syn1Cre$^{+/-}$IFNAR$^{fl/fl}$ (n=6) and Cre$^{-/-}$IFNAR$^{fl/fl}$ mice (n=6) as well as Tam-treated Sall1CreER$^{+/-}$IFNAR$^{fl/fl}$ (n=6) and Sall1CreER$^{-/-}$IFNAR$^{fl/fl}$ mice (n=9). Shown are all data points with means. Each data point represents the percentage of immunopositive cells of one mouse.

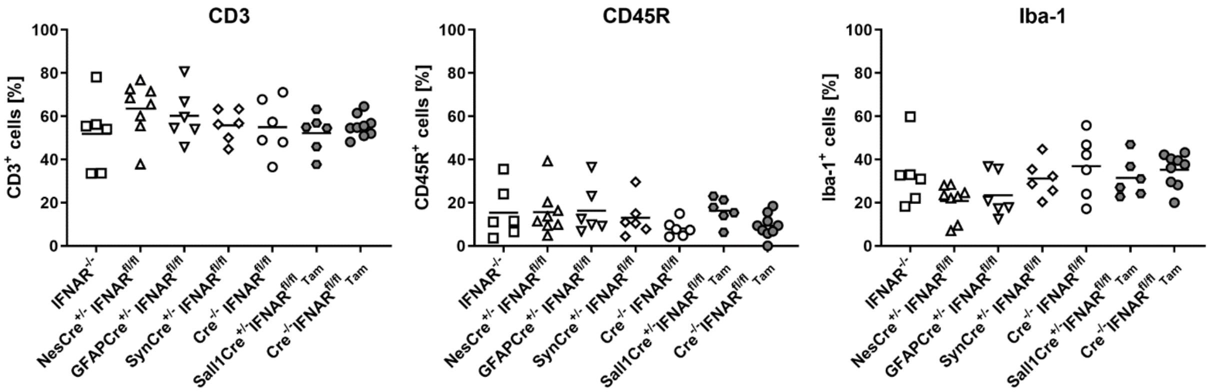

Fig. S5: GFAP$^+$, MAC3$^+$, and TMEM119$^+$ cells in the hippocampus at 4 days after TMEV-BeAn infection. No significant differences in the semiquantitative analysis of GFAP$^+$, MAC3$^+$ and TMEM119$^+$ cells were found between TMEV-infected IFNAR$^{-/-}$, NesCre$^{+/-}$IFNAR$^{fl/fl}$, GFAPCre$^{+/-}$IFNAR$^{fl/fl}$, Syn1Cre$^{+/-}$IFNAR$^{fl/fl}$ and Cre$^{-/-}$IFNAR$^{fl/fl}$ mice (n=5-10). Shown are all data points with means. Each data point represents the semiquantitative score of immunopositive cells of one mouse.

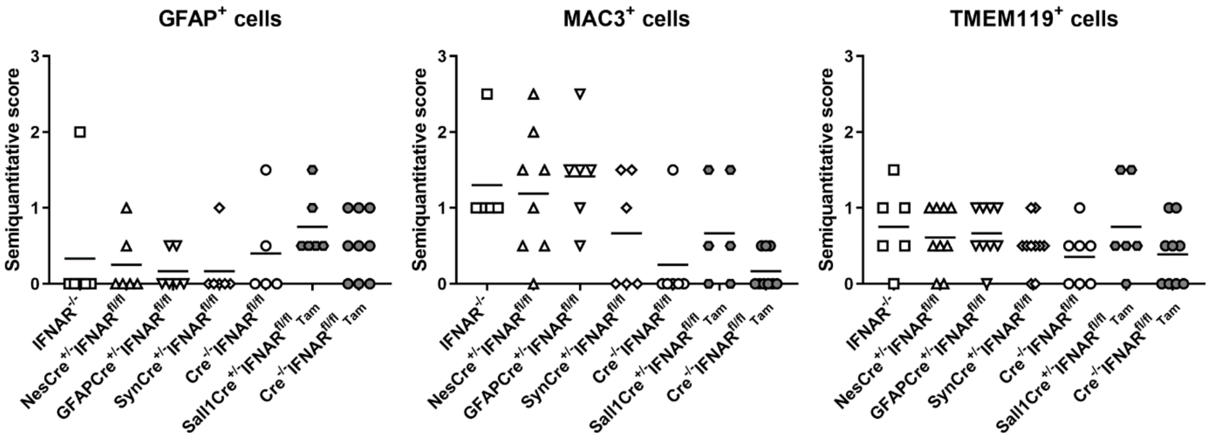

Fig. S6: A plaque assay confirms the presence of infectious virus in the brain at 4 days after TMEV-BeAn infection. Infectious virus was found in TMEV-infected IFNAR$^{-/-}$, NesCre$^{+/-}$IFNAR$^{fl/fl}$, GFAPCre$^{+/-}$IFNAR$^{fl/fl}$, Syn1Cre$^{+/-}$IFNAR$^{fl/fl}$ and Cre$^{-/-}$IFNAR$^{fl/fl}$ mice as well as Tam-treated Sall1CreER$^{+/-}$IFNAR$^{fl/fl}$ and Sall1CreER$^{-/-}$IFNAR$^{fl/fl}$ mice. Shown are all data points with means. Each data point represents the plaque-forming units/ml of one mouse (n=3).

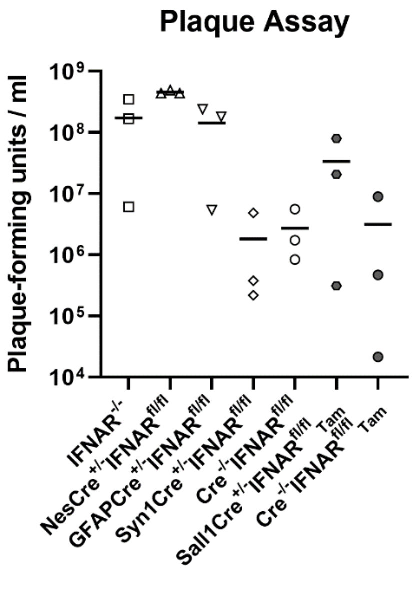

Fig. S7: Nonparametric Spearman correlation coefficients of RT-qPCR data of the cerebrum at 4 days after TMEV-BeAn infection.

	TMEV	*Ifnb1*	*Isg15*	*Eif2ak1*	*Tnfa*	*Il1b*	*Il6*	*Il10*	*Il12b*	*Ifng*
TMEV	1.00	0.66	0.16	-0.01	0.88	0.72	0.78	0.43	0.71	0.51
Ifnb1	0.66	1.00	0.01	-0.10	0.63	0.56	0.60	0.33	0.53	0.45
Isg15	0.16	0.01	1.00	0.89	0.38	0.14	0.38	0.60	0.48	0.60
Eif2ak1	-0.01	-0.10	0.89	1.00	0.17	-0.04	0.16	0.45	0.28	0.48
Tnfa	0.88	0.63	0.38	0.17	1.00	0.76	0.86	0.52	0.78	0.68
Il1b	0.72	0.56	0.14	-0.04	0.76	1.00	0.73	0.35	0.47	0.30
Il6	0.78	0.60	0.38	0.16	0.86	0.73	1.00	0.55	0.73	0.66
Il10	0.43	0.33	0.60	0.45	0.52	0.35	0.55	1.00	0.61	0.74
Il12b	0.71	0.53	0.48	0.28	0.78	0.47	0.73	0.61	1.00	0.73
Ifng	0.51	0.45	0.60	0.48	0.68	0.30	0.66	0.74	0.73	1.00

1.0
0.5
0
-0.5
-1.0

Fig. S8: Nonparametric Spearman correlation coefficients of TMEV RNA and cytokine array data of the cerebrum at 4 days after TMEV-BeAn infection.

	TMEV	IFN-α	IFN-β	IFN-γ	TNF	IL-1β	IL-6	IL-10	IL-12	CCL-2	CCL-5	CXCL-1	CXCL-10
TMEV	1.00	0.70	0.69	0.09	0.38	0.59	0.61	0.25	0.10	0.68	0.64	0.67	0.53
IFN-α	0.70	1.00	0.81	0.30	0.54	0.79	0.86	0.58	0.21	0.85	0.81	0.71	0.78
IFN-β	0.69	0.81	1.00	0.19	0.54	0.69	0.79	0.45	0.24	0.73	0.74	0.64	0.60
IFN-γ	0.09	0.30	0.19	1.00	0.46	0.28	0.32	0.55	0.45	0.28	0.35	0.28	0.23
TNF	0.38	0.54	0.54	0.46	1.00	0.42	0.39	0.60	0.37	0.31	0.30	0.25	0.31
IL-1β	0.59	0.79	0.69	0.28	0.42	1.00	0.84	0.62	0.22	0.88	0.83	0.75	0.81
IL-6	0.61	0.86	0.79	0.32	0.39	0.84	1.00	0.56	0.21	0.89	0.91	0.82	0.72
IL-10	0.25	0.58	0.45	0.55	0.60	0.62	0.56	1.00	0.27	0.48	0.51	0.47	0.46
IL-12	0.10	0.21	0.24	0.45	0.37	0.22	0.21	0.27	1.00	0.08	0.09	0.05	0.16
CCL-2	0.68	0.85	0.73	0.28	0.31	0.88	0.89	0.48	0.08	1.00	0.93	0.86	0.84
CCL-5	0.64	0.81	0.74	0.35	0.30	0.83	0.91	0.51	0.09	0.93	1.00	0.82	0.80
CXCL-1	0.67	0.71	0.64	0.28	0.25	0.75	0.82	0.47	0.05	0.86	0.82	1.00	0.65
CXCL-10	0.53	0.78	0.60	0.23	0.31	0.81	0.72	0.46	0.16	0.84	0.80	0.65	1.00

1.0
0.5
0
-0.5
-1.0

Fig. S9: TMEV antigen and IL-6 and IL-10 protein expression in the brain of a TMEV-infected NesCre$^{+/-}$IFNAR$^{fl/fl}$ mouse. Shown is the hippocampus of a TMEV-infected NesCre$^{+/-}$IFNAR$^{fl/fl}$ mouse at 4 days post infection (serial sections of the animal shown in Fig. 3). TMEV: Note high amount of TMEV antigen in the hippocampus. IL-6: There is a strong IL-6 expression by hippocampal neurons (upper inset), whereas most perivascular mononuclear cells lack an IL-6 immunoreaction (lower inset). IL-10: IL-10 immunoreactivity was only found in few small round and elongated cells (upper inset) as well as medium-sized round cells (arrow; lower inset). Immunohistochemistry using the avidin-biotin-peroxidase complex method with the chromogen 3'3-diaminobenzidine and Mayer's hematoxylin counterstaining (serial sections).

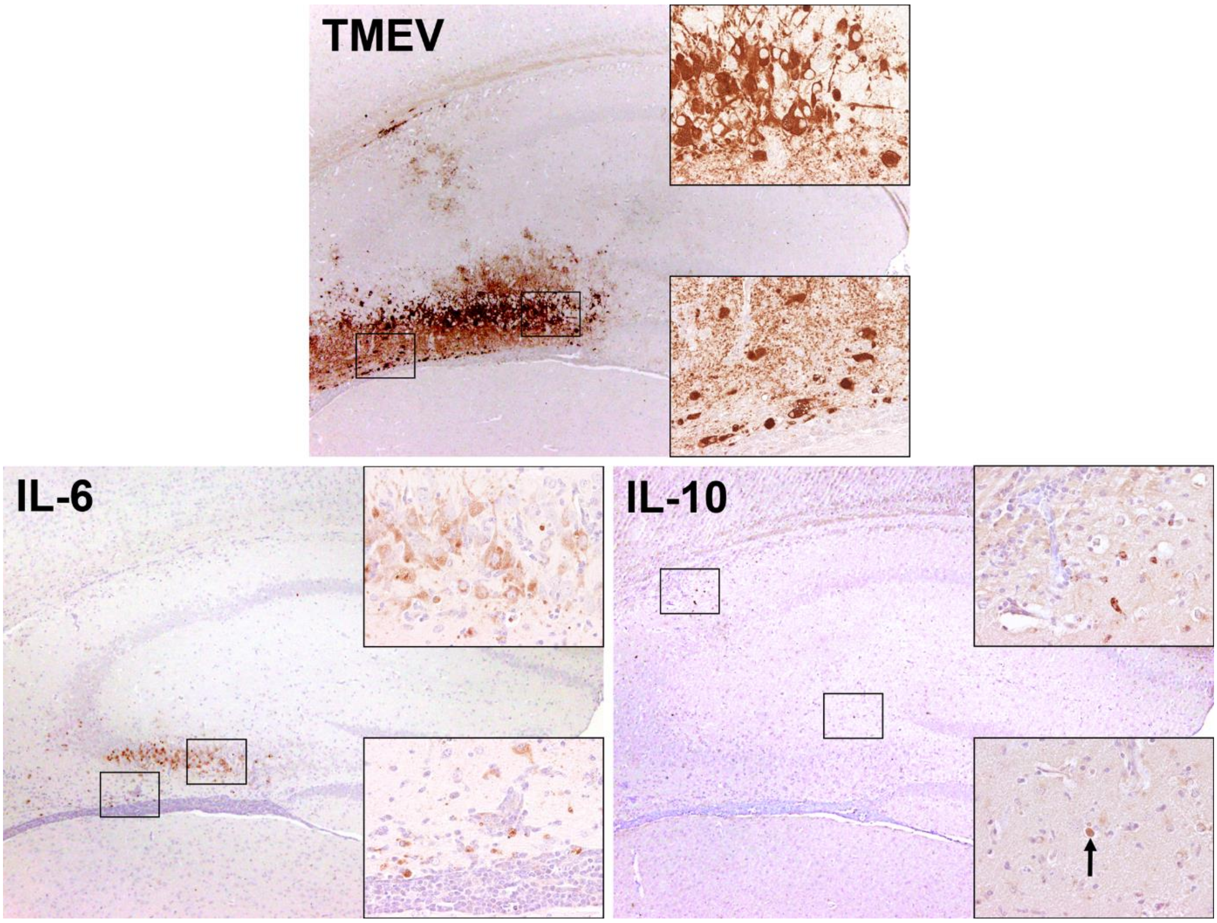

Supplement: Supplementary file 1 — Additional file 1: Table S1. Summary of primer pairs used for reverse transcriptase polymerase chain reaction. Table S2. Transcriptional changes in the cerebrum of TMEV- and mock-infected SJL and C57BL/6 mice (fold changes). Fig. S1. RT-qPCR of the cerebrum at 4, 7 and 14 days after TMEV-BeAn infection (dpi). lfna mRNA transcripts were downregulated in TMEV- compared to mock-infected SJL and C57BL/6 mice at 14 dpi, whereas no significant impact of TMEV infection on Ifnb1 mRNA transcripts was found. Irf7, Isg15 and Eif2ak1 mRNA transcripts were increased in TMEV- compared to mock-infected SJL and C57BL/6 mice at 4, 7 and 14 dpi except for Isg15 mRNA transcripts in C57BL/6 mice at 14 dpi. Mock-infected SJL: n=5 (4, 7, 14 dpi); TMEV-infected SJL: n=6 (4,7 dpi), n=5 (14 dpi); mock-infected C57BL/6 mice: n=6 (4, 14 dpi), n=5 (7 dpi); TMEV-infected C57BL/6 mice: n=4 (4 dpi), n=3 (7 dpi), n=5 (14 dpi). The mRNA copy numbers were normalized using a normalization factor calculated from three housekeeping genes (Gapdh, Actb, Hprt). Mann Whitney tests: * p < 0.05; ** p < 0.01. Shown are all data points with means. Each data point represents the mRNA copy number of one mouse. Fig. S2. ISG15, OAS1 and PKR protein expression in the brain of TMEV-infected C57BL/6 mice. Shown is the hippocampus of TMEV-infected C57BL/6 mice at 4 (ISG15 and PKR) and 7 (OAS1) days post infection. Immunohistochemistry using the avidin-biotin-peroxidase complex method with the chromogen 3’3-diaminobenzidine and Mayer’s hematoxylin counterstaining. Fig. S3. Hippocampal cell loss at 4 days after TMEV-BeAn infection. No significant differences were found between TMEV-infected IFNAR-/- (n=6), NesCre+/-IFNARfl/fl (n=10), GFAPCre+/-IFNARfl/fl (n=9), Syn1Cre+/-IFNARfl/fl (n=10) and Cre-/-IFNARfl/fl mice (n=7) as well as Tam-treated Sall1CreER+/-IFNARfl/fl (n=6) and Sall1CreER-/-IFNARfl/fl mice (n=9). Shown are all data points with means. Each data point represents the semiquantitative score of one mouse. Fig. [file 12974_2023_2737_MOESM1_ESM.pdf]
